# The Dual‐Active‐Site Catalysts Containing Atomically Dispersed Pr^3+^ with Ni/CeO_2_ for CO_2_ Hydrogenation to Methane

**DOI:** 10.1002/smll.202504707

**Published:** 2025-06-11

**Authors:** Neha Choudhary, Navdeep Srivastava, Harshini V. Annadata, Biplab Ghosh, Patrick Da Costa

**Affiliations:** ^1^ Institut Jean Le Rond D'Alembert Sorbonne Université CNRS UMR 7190, 2 Place de la Gare de Ceinture Saint‐Cyr‐L'Ecole 78210 France; ^2^ Department of Chemistry Indian Institute of Technology‐Indore Simrol Khandwa Road 453552 India; ^3^ Beamline Development and Application Section Bhabha Atomic Research Center Mumbai 400085 India

**Keywords:** CO_2_ methanation, interfacial sites, Ni catalyst, oxygen vacancy, single‐atom catalysts

## Abstract

In this study, uniformly dispersed Pr^3+^ as an isolated atom over Ni/CeO_2_ catalyst (Ni‐Pr/CeO_2_) is designed to enhance catalytic activity for CO_2_ methanation, achieving an impressive 87% conversion with ≈100% CH_4_ selectivity at 300 °C temperature. In contrast, the traditional Ni/CeO_2_ and NiPr/CeO_2_‐imp catalysts exhibit poor conversion and selectivity, highlighting the proof of concept on the advantage of atomic‐scale dispersion. Structural analysis via PXRD, XAS, and XPS confirms the successful incorporation of Pr^3+^ into the CeO_2_ lattice by creating defects. XPS and XAS studies further reveal a significant increase in oxygen vacancies, a key factor in enhancing catalytic performance at lower reaction temperatures. STEM‐EDS analysis confirms the ultra‐dispersion of Pr^3+^ (≈7 wt.%) over CeO_2_, ensuring a highly active catalyst surface. H_2_‐TPR and CO_2_‐TPD results suggest that the Pr^3+^ doping enhances the catalytic activity by decreasing the reduction temperature and increasing basic sites. Additionally, long‐term stability tests demonstrate no significant loss in activity over 40 h, confirming the catalyst's robustness and recyclability. This work provides critical insights into the structure‐activity relationship of Pr^3+^‐modified Ni/CeO_2_ catalysts, emphasizing the role of oxygen vacancies in optimizing CO_2_ hydrogenation efficiency.

## Graphical abstract

Herein, the Ni‐Pr/CeO_2_ catalyst is reported for an efficient catalytic conversion of CO_2_ to methane with 87% conversion and 100% selectivity. STEM‐EDS analysis reveals the uniform dispersion of Pr^3+^ ions on CeO_2_ support. Doping of Pr^3+^ over CeO_2_ generates oxygen vacancies, which leads to excellent catalytic performance with long‐run activity with no significant loss in activity.

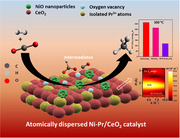

## 1 Introduction

In recent years, the emission of carbon dioxide (CO_2_) from traditional fossil fuels has impacted the global climate and ecosystem.^[^ ^1^, ^2^ ^]^ Hence, the conversion of CO_2_ into value‐added chemicals, including methanol, ethanol, methane, and long‐chain hydrocarbons, has drawn attention.^[^ ^3^, ^4^ ^]^ CO_2_ conversion into synthetic renewable energy alternatives using renewable H_2_ is a promising strategy to deal with the issue of CO_2_ emission.^[^ ^5^, ^6^ ^]^ In this regard, the thermal catalytic approach of CO_2_ to methane conversion over photo‐ and electro‐catalytic approach is more sustainable and practical for industrial application.^[^ ^7^, ^8^, ^9^ ^]^ For selective CO_2_ methanation, a low‐temperature reaction is more favorable to avoid the CO formation over methane from CO_2_. The methanation process is exothermic, and at high temperature, the selectivity for methane as a product decreases due to thermodynamics.^[^ ^10^, ^11^, ^12^ ^]^

Since, at low‐reaction temperatures, high methane production is difficult due to kinetic limitations,^[^ ^10^, ^13^ ^]^ various noble metals and non‐noble metals have been explored and utilized for methane production.^[^ ^14^, ^15^ ^]^ Rh, Ru, Ag, Pd, and Pt‐based catalysts have shown excellent activity with good selectivity at low reaction temperatures.^[^ ^16^, ^17^, ^18^ ^]^ However, their high cost limits their large‐scale application by making the overall process expensive. On the other hand, Ni‐based catalysts have drawn great attention owing to their low cost and good catalytic activity. Although, they usually suffer from poor catalytic performance at temperatures less than 300 °C.

To deal with this issue, various strategies have been opted by varying synthetic methods, amount of active metal or dopant, support materials (metal oxides, MOFs, carbon‐based material), and tuning shape or morphology, etc, to achieve high conversion and selectivity at low reaction temperature.^[^ ^19^, ^20^, ^21^ ^]^ It was reported that Ni nanoparticles supported over metal oxides showed catalytic performance owing to their enhanced oxygen vacancies, improved basic sites, and, consequently, increased hydrogen spillover.^[^ ^22^, ^23^, ^24^ ^]^ Ni nanoparticles (NPs) over ceria support demonstrated to be good catalysts due to the formation of interfacial sites, which maximize the synergistic effect between existing metals.^[^ ^25^, ^26^ ^]^

To improve the catalytic activity at low reaction temperatures for tuning their electronic properties, the doping of active metal has been demonstrated. The doping of active metal with Ni over ceria could enhance the surface oxygen mobility by creating more Ce^3+^ ions on the surface, which as a result, supports the formate pathway during the reaction and promotes the selectivity toward methane as a product.^[^ ^27^, ^28^ ^]^ Xu et al. demonstrated that incorporating Cr^3+^ into CeO_2_ enhances the formation of surface oxygen vacancies, Ce^3+^ species, and hydroxyl groups, thereby facilitating the formate‐mediated pathway for methane production at low temperatures.^[^ ^29^ ^]^ Moreover, Y‐doped CeO_2_ with Ni nanoparticles were prepared by Sun et al. and they observed that the oxygen vacancies were highly affected by the particle size of Ni and improved the metal‐support interaction.^[^ ^30^ ^]^ Additionally, Ru single atoms were doped over ceria support and impregnated with Ni NPs where Zhang et al. observed that Ru_1_ SACs were able to convert CO_2_ to CO, and Ni NPs boosted the sequential conversion to methane product.^[^ ^7^ ^]^ They concluded that the simultaneous presence of Ru_1_ and Ni sites significantly enhances the overall reaction performance.

Lanthanide doping over ceria with Ni NPs was also reported for enhanced catalytic performance. Liu et al. prepared a series of Sm‐doped CeO_2_ supports with varying Samarium loading to optimize the metal‐support interaction in Ni‐based catalysts. Their findings indicated that Ni/Sm_0.25_Ce_0.75_O_2−δ_, showed excellent catalytic performance, which exhibited the highest metallic Ni surface area and the largest active interface.^[^ ^31^ ^]^ Eu^3+^ doping promoted Ni/CeO_2_ catalyst was also analyzed where they observed the more interfacial sites improved the catalytic performance by promoting bidentate carbonate formation and consequently leading to faster hydrogenation at low‐temperature.^[^ ^10^ ^]^ Similarly, Ni/La‐CeO_2_ was prepared by Zhang et al., where the incorporation of La species into the CeO_2_ support, followed by calcination at 600 °C (CeO_2_‐La‐600), led to the formation of a La‐Ce‐O solid solution with a thin La_2_O_2_CO_3_ layer on the surface, resulting in an increased number of basic sites and oxygen vacancies.^[^ ^32^ ^]^ This solid solution promoted the adsorption and dissociation of CO_2,_ which led to enhanced catalytic performance. Moreover, mixed metal oxides of Sm^3+^, Pr^3+,^ and Mg^2+^ cations over Ni/CeO_2_ catalyst were prepared by Siakavelas et al.^[^ ^33^ ^]^ for comparing them for methanation, and it was observed that Ni/Pr‐Ce showed superior catalytic activity over other synthesized materials. It was observed that the doping of Pr^3+^ possesses more oxygen vacancies than Sm and Mg cations, and the presence of Pr_2_O_3_‐CeO_2_ also enhances the dispersion of Ni sites by restricting agglomeration.

On the basis of previous reports, we synthesized uniformly dispersed Pr doped over CeO_2_ and decorated with Ni nanoparticles (named Ni‐Pr/CeO_2_), where first atomically dispersed Pr/CeO_2_ was prepared using the co‐precipitation method and further covered with Ni nanoparticles for enhanced catalytic activity. The presence of both Ni and Pr promoted the catalytic performance owing to the synergistic effect between the metals. Also, when this material was compared with an undoped Ni/CeO_2_ catalyst then, the Ni‐Pr/CeO_2_ showed superior catalytic performance with excellent selectivity toward methane as a product. The catalyst is extensively characterized via various techniques like XPS, STEM‐HAADF, EXAFS, XANES, H_2_‐TPR, CO_2_‐TPD, and H_2_‐TPD. XPS analysis confirms the increase in oxygen vacancies after doping Pr^3+,^ whereas STEM‐EDX confirms the uniform dispersion of Pr^3+^ ions over ceria support. The catalyst was tested for methane production where at 300 °C temperature, Ni‐Pr/CeO_2_ showed excellent catalytic conversion, i.e., 87% with 100% selectivity. These results confirm that the doping of Pr^3+^ over ceria with Ni nanoparticles plays an important role and provides more active sites. The effect of high oxygen mobility and basic sites was also analyzed in this study.

## 2 Results and Discussion

### 2.1 Structural Characterizations of Catalysts

In order to analyze the metal loadings of Ni and Pr over ceria, ICP‐OES was performed (Table S1, Supporting Information). As expected, the Pr and Ni loading in Ni‐Pr/CeO_2_ catalysts was found to be 8 and 7 wt.%, respectively.

**Figure 1:**
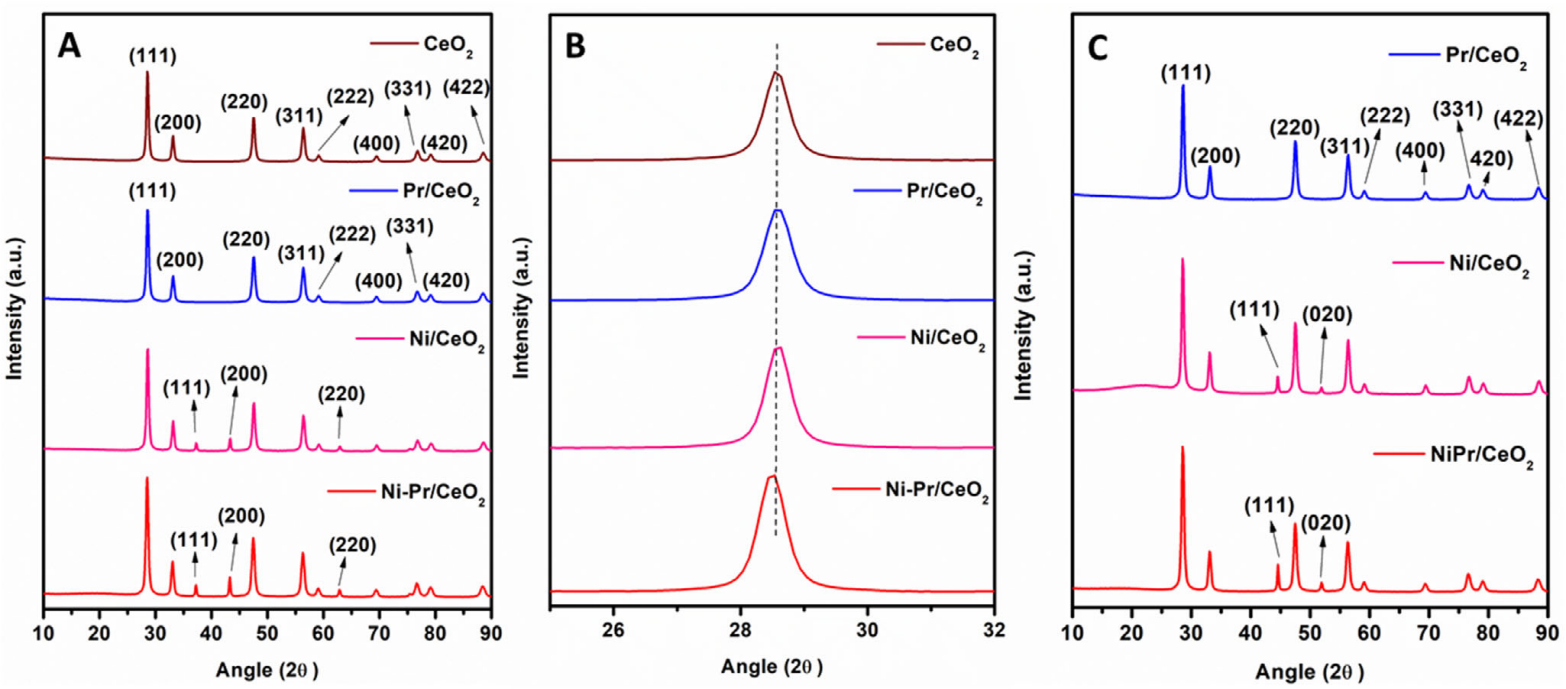
PXRD of A) fresh catalysts, B) shifting of (111) plane of CeO_2_ after incorporation of NiO and Pr, and C) reduced catalysts.

X‐ray diffractograms of both fresh and reduced CeO_2_, Pr/CeO_2_, Ni‐Pr/CeO_2,_ and Ni/CeO_2,_ as reported in **Figure** **1**. All the catalysts showed typical peaks of fluorite structure of CeO_2_ with diffraction peaks at 28.89°, 33.13°, 47.51°, 56.31°, 59.1°, 69.47°, 76.73°, 78.98° and 88.44° corresponds to (111), (200), (220), (311), (222), (400), (331), (420) and (422) planes in agreement with JCPDS# 43–1002.^[^ ^27^, ^34^ ^]^ As indicated in Figure 1A, there was no prominent peak of Pr was observed in the case of fresh Pr/CeO_2_ and Ni‐Pr/CeO_2_, due to low Pr loading and similar crystal lattice of Pr and Ce.^[^ ^35^, ^36^ ^]^ Further, the weak diffraction peaks of NiO were observed in the case of fresh Ni/CeO_2_ and Ni‐Pr/CeO_2_ at 37.36°, 43.6° and 63.04° for (111), (200) and (220) planes confirmed with JCPDS# 44–1159.^[^ ^30^, ^37^ ^]^ When the major diffraction plane of CeO_2_ (111) was enlarged to compare, there was a shift in the case of Ni‐Pr/CeO_2_ was observed toward a lower 2θ value, which confirms the incorporation of Pr^3+^ ion in ceria lattice (Figure 1B).^[^ ^36^, ^38^ ^]^ This shift in d_(111)_ spacing may be caused by the incorporation of Pr^3+^ cations (1.27 Å) in the place of Ce^4+^ (0.97 Å) ions in typical CeO_2_ fluorite structure, and as a result, lattice expansion leads to significant decrease in 2θ value.^[^ ^33^, ^39^, ^40^ ^]^ This hypothesis of lattice expansion of CeO_2_ can be evidenced by the crystallite size calculations of fresh and reduced catalysts for the d_(111)_ plane of ceria (Table S2, Supporting Information). The full‐width half maxima (FWHM) for undoped CeO_2_ and Ni/CeO_2_ were lower than the doped Ni‐Pr/CeO_2_ for CeO_2_ (111) plane, representing higher crystallite size for CeO_2_ than Ni‐Pr/CeO_2_ and Pr/CeO_2_ catalysts. For reduced Ni/CeO_2_ and Ni‐Pr/CeO_2_ catalysts, the diffraction peaks at 44.4° and 52.09° correspond to (111) and (020) planes for Ni(0) observed in agreement with JCPDS# 04–0850 as shown in Figure 1C.^[^ ^10^, ^41^ ^]^ Similar to the fresh catalyst, in the case of the reduced catalyst, Ni/CeO_2_ has a higher crystallite size than the Ni‐Pr/CeO_2_ catalyst, indicating that the doping of Pr^3+^ ions enhances the dispersion of Ni species over ceria by inhibiting the growth of ceria and NiO. Additionally, for comparison, the NiPr/CeO_2_‐imp catalyst was also analyzed, as depicted in Figure S1 (Supporting Information). The peaks of the fluorite structure of CeO_2_ were observed in the case of both fresh and reduced catalysts. Although the fresh NiPr/CeO_2_‐imp catalyst displayed peaks of NiO corresponding to JCPDS# 44–1159, the reduced catalyst showed the peaks of Ni(0) metallic as expected. There was no obvious peak of Pr observed in both fresh and reduced catalysts.

N_2_ adsorption–desorption analysis was performed to estimate the surface area, pore size, and pore volume (Figure S2, Supporting Information). The results are in good agreement with PXRD results, where the incorporation of Pr^3+^ ions enhanced the surface area from 23 to 30 m^2^ g^−1^ in Ni‐Pr/CeO_2_ catalyst when compared with Ni/CeO_2_. On the other hand, the impregnation of Ni over Pr/CeO_2_ decreases the surface area from 37 to 30 m^2^ g^−1^ in NiPr/CeO_2_‐imp. The doping of Pr^3+^ ions generates defects on the surface of CeO_2_ and creates more surface vacancies, which leads to a higher surface area of Ni‐Pr/CeO_2_ than Ni/CeO_2_.^[^ ^10^ ^]^ Additionally, this increase in surface area in the case of Ni‐Pr/CeO_2_ is in good agreement with the crystallite size as mentioned in Table S2 (Supporting Information) that as the crystallite size decreases, the surface area increases.^[^ ^42^, ^43^ ^]^ As shown in Figure S2A (Supporting Information), adsorption–desorption isotherms displayed type‐IV isotherm with H4 hysteresis loops, confirming the mesoporous structure of the catalysts.^[^ ^44^, ^45^ ^]^ This result of the presence of mesopore is further confirmed by BJH pore size distribution values in Figure S2B (Supporting Information), which ranges from 10–30 nm pore diameter. All catalysts have similar patterns in pore size distribution and BET desorption isotherms, maybe owing to having similar support material in the dominant amount.

**Figure 2:**
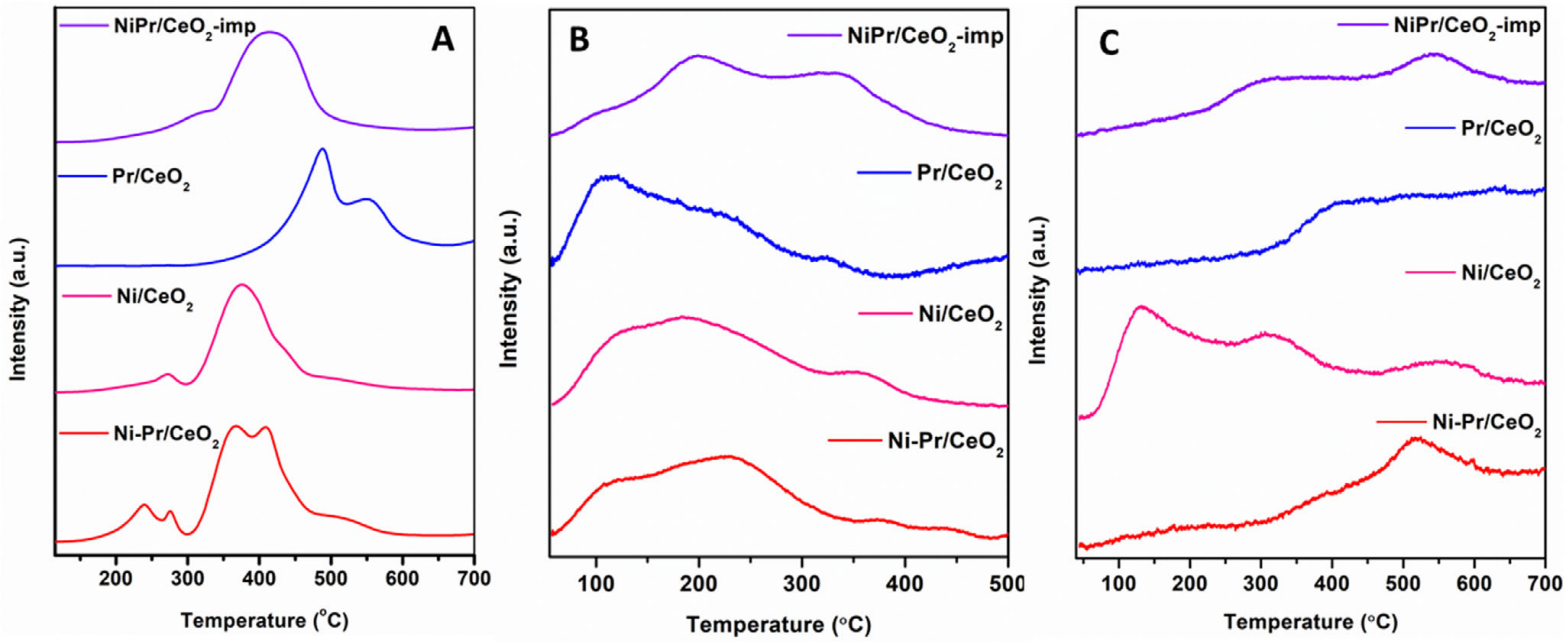
A) H_2_‐TPR, B) CO_2_‐TPD, and C) H_2_‐TPD profile for all the catalysts.

To analyze the reduction of the catalysts, H_2_ TPR analysis was performed as shown in **Figure** **2**A. Undoped CeO_2_ support showed three peaks in a broad temperature range from 350–580 °C, i.e., 357, 420, and 548 °C, indicating the removal of surface oxygen with a reduction of Ce^4+^ to Ce^3+^.^[^ ^10^, ^46^ ^]^ When Pr/CeO_2_ was analyzed where two major peaks were observed at higher temperatures, i.e., 488 and 551 °C, indicating the reduction of oxygen species from Pr‐Ce mixed oxide. In the case of Ni/CeO_2_, Ni‐Pr/CeO_2_, and NiPr/CeO_2_‐imp catalysts, the minor peaks between 230–300 °C represent the reduction of reactive surface oxygen on ceria support.^[^ ^28^, ^47^ ^]^ Further, the broad peak between 300–450 °C corresponds to the reduction of strongly bounded NiO clusters was observed.^[^ ^48^ ^]^ It was reported that based on the size of NiO nanoparticles, the reduction temperature was highly influenced. Nevertheless, it was assumed that the removal of surface oxygen occurs in a similar temperature range (200–300 °C), which might be affecting the reduction peaks of NiO in the case of Pr^3+^ doped catalyst.^[^ ^49^, ^50^ ^]^ It was observed that the Pr‐doped catalyst displayed a major reduction peak from 300 to 450 °C temperature at a comparatively lower temperature than the undoped Ni/CeO_2_ catalyst. This data revealed that the doping of Pr^3+^ might enhance the metal support interaction between NiO and ceria support.^[^ ^35^, ^51^ ^]^ The split peak broadening in Ni‐Pr/CeO_2_ confirmed that the doping of Pr^3+^ increases the oxygen vacancies on ceria support.^[^ ^10^, ^28^ ^]^ In the case of impregnated NiPr/CeO_2_‐imp catalyst, the size of Ni nanoparticles is expected to be high, which leads to the weak interaction between NiO and ceria. Consequently, a major broad peak at 400 °C was observed.^[^ ^28^, ^52^ ^]^ Additionally, the reduction peak at higher temperatures (450–550 °C) in Ni‐Pr/CeO_2_ catalyst indicates the doping of Pr^3+^ into the lattice of CeO_2_ support owing to enhanced reducibility of CeO_2_ support, and this can be compared with Pr/CeO_2_ catalyst.^[^ ^28^, ^53^ ^]^ This result is also confirmed with H_2_ consumption calculation which indicates that the H_2_ consumption of the Ni‐Pr/CeO_2_ catalyst was higher than that of the Ni/CeO_2_ catalyst, i.e., 163 and 104 µmol g^−1^ respectively. In summary, the doping of Pr^3+^ improves the oxygen vacancy as well as the reducibility of the CeO_2_ support.

To analyze the basic sites of the catalyst, CO_2_‐TPD was performed for all the catalysts, as reported in Figure 2B. The surface basicity plays an important role in determining the CO_2_ dissociation and hydrogen activity of the catalyst.^[^ ^54^, ^55^ ^]^ Based on the desorption curve, the temperature range was divided into three major regions, i.e., 55–150 °C, 150–300 °C, and 300–500 °C corresponds to I, II, and III types. The basic sites were calculated using the area under the curve values for each of the samples (**Table** **1**). The total basic sites were obtained highest in Ni‐Pr/CeO_2_ than Ni/CeO_2_ and impregnated NiPr/CeO_2_‐imp catalysts. Basic sites between the 300–500 °C (type III) temperature range play an important role in CO_2_ adsorption–desorption and, consequently, influence the catalytic activity.^[^ ^56^, ^57^ ^]^ As mentioned in Table 1, type III basic sites are surprisingly high in the case of Ni‐Pr/CeO_2_ catalyst, indicating that the doping of Pr^3+^ enhances the basic sites, which is in good agreement with H_2_‐TPR results.

**Table 1:** The distribution of basic sites is estimated from the CO_2_ TPD profile.

Catalyst	Basic sites [µmol g^−1^]			
	I (55–150 °C)	II (150–300 °C)	III (300–500 °C)	Total
Ni/CeO_2_	2.15	11.08	4.25	17.43
Ni‐Pr/CeO_2_	4.17	10.51	36.61	51.29
NiPr/CeO_2_‐imp	1.79	12.03	7.09	20.92

H_2_‐TPD analysis was also performed for all the catalysts to analyze the hydrogen desorption affinity of the catalyst (Figure 2C). All Ni‐based catalysts mainly showed three peaks, i.e., below 250 °C, 250–400 °C, and above 400 °C. The peak intensity in all three catalysts varies based on their interaction with metal support. In Pr/CeO_2_, the peak below 300 °C corresponds to desorbed hydrogen from Ni metal was absent and can be comparable with other Ni‐based catalysts. The intense peaks at low temperatures below 250 °C in Ni/CeO_2_ catalyst showed the desorption of hydrogen from active metal species, confirming the strong interaction between Ni and ceria support.^[^ ^7^ ^]^ The peak at higher temperature in all three Ni‐based catalysts above 400 °C confirmed the strong interaction between Pr‐Ni with ceria support, which is absent in the case of Pr/CeO_2_. This also confirms that the synergistic effect of Ni and Pr improves the hydrogen desorption in catalysts. In both Ni‐Pr/CeO_2_ and NiPr/CeO_2_‐imp catalysts, a broad peak above 250–400 °C showed hydrogen desorption from ceria support. This could be hydrogen spillover due to the presence of Pr^3+^.^[^ ^7^, ^10^, ^47^ ^]^ This result is in agreement with the H_2_‐TPR result, where Pr^3+^ doping over ceria support enhances the reducibility and H_2_ spillover over, creating oxygen vacancy.^[^ ^28^, ^58^ ^]^ Overall, doping of Pr^3+^ with the presence of Ni nanoparticles could improve the chemisorption of hydrogen over the catalyst surface.

**Figure 3:**
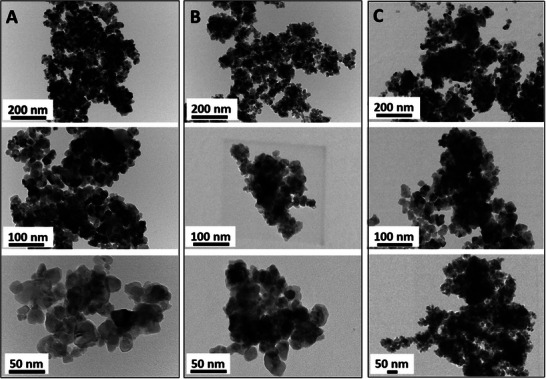
HR‐TEM images of A) Ni/CeO_2_, B) Pr/CeO_2,_ and C) Ni‐Pr/CeO_2_ catalysts at 200, 100, and 50 nm magnification.

Additionally, the morphological analysis was performed on reduced samples using High‐resolution Transmission electron microscope (HR‐TEM) analysis (**Figure** **3**). In all the catalysts, lattice fringes of CeO_2_ support can clearly be seen in the inset image in Figure 3 for reduced Pr/CeO_2,_ Ni/CeO_2_, and Ni‐Pr/CeO_2_ catalysts. However, it was difficult to analyze the particle size from the HR‐TEM images owing to the similar contrast of Ni and ceria.^[^ ^7^, ^59^, ^60^ ^]^

**Figure 4:**
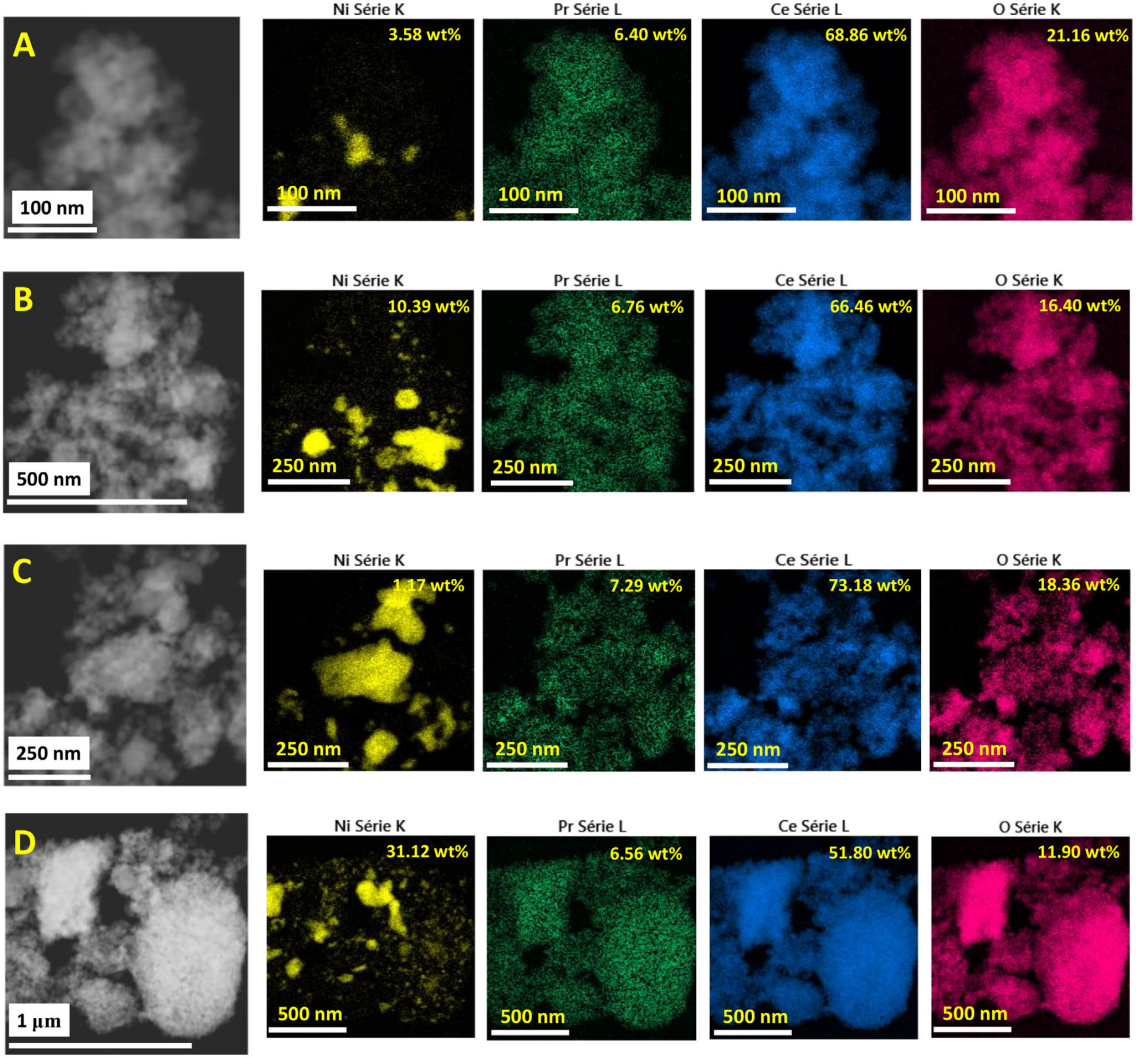
STEM mapping of Nickel, Praseodymium, Cerium, and Oxygen elements of Ni‐Pr/CeO_2_ catalyst at different regions at higher magnifications with scale bars of A) 100 nm, B) 250 nm, C) 250 nm, and D) 500 nm.

Aberrated corrected scanning transmission electron microscopy (AC‐STEM) imaging and elemental analysis EDX analysis were performed to confirm the presence of Pr, Ni, Ce, and O elements (**Figure** **4**; Figures S3–S5, Supporting Information). AC‐STEM in annular dark field (ADF) mode was performed for Pr/CeO_2_, where no cluster of Pr was observed_,_ confirming the dispersion of Pr over ceria as shown in Figure S3 (Supporting Information) and due to microscopic limitations and similar atomic numbers of Pr and Ce, Pr atoms cannot be observed.^[^ ^1^, ^23^ ^]^ The HAADF images and EDX analysis with color mapping were performed at different magnifications at different regions of Pr/CeO_2_ catalysts to analyze the uniform dispersion of Pr over ceria. The results revealed that in each elemental map, the distribution of Pr over ceria was found to be uniform, i.e., ≈8 wt.%, which was in good agreement with ICP‐OES results (Table S1, Supporting Information). As shown in Figure 4, Ni‐Pr/CeO_2_ shows ≈7 wt.% of Pr with varied amounts of Ce, Ni, and O. The variation in the amount of Ni and Ce could be justified due to the presence of their oxides in the catalyst. It is also confirmed with EDX analysis that Ni NPs and Pr atoms are not overlapped and are well dispersed over ceria support. In all the elemental maps, the amount of Pr was found to be consistent, which was again confirmed with ICP‐OES results. In summary, it was observed that the Pr is uniformly dispersed over CeO_2,_ which is consistent even after the impregnation of NiO NPs. For comparison, Ni/CeO_2_ and NiPr/CeO_2_‐imp catalysts were also analyzed using STEM‐EDX analysis. In both cases, the morphology of particles was quite distinct and not consistent (Figures S4 and S5, Supporting Information). Also, the amount of Ni and Pr was not consistent even at low magnification, i.e., 100 nm. In EDX analysis of Ni/CeO_2_ and NiPr/CeO_2_‐imp impregnated catalyst, it is revealed that both Ni and Pr wt.% were varied with different magnifications, which could be related to the presence of their oxides and in agglomerated form. This further confirms the importance of the precise synthesis of Ni‐Pr/CeO_2_ uniformly dispersed catalysts.

**Figure 5:**
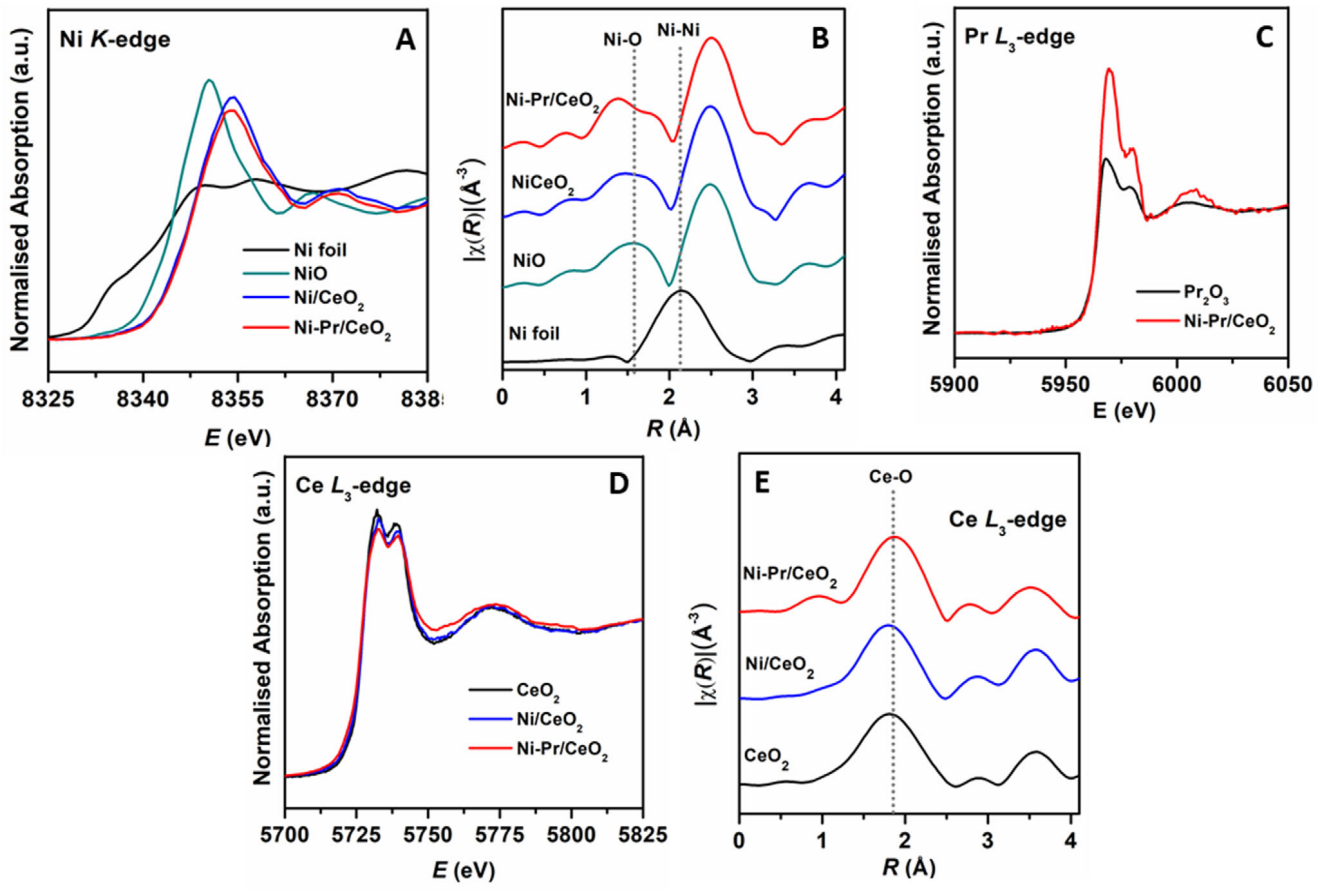
A,B) Normalized XANES Ni K‐edge with Fourier transform spectra, C)Pr L_3_‐edge XANES, D,E) Ce L_3_‐edge XANES, and Fourier transform spectra.

The local coordination environment and oxidation states of Ni, Pr, and Ce were analyzed using Ni K‐edge, Ce L_3_‐edge, and Pr L_3_‐edge X‐ray Absorption Near‐Edge Structure (XANES) and Extended X‐ray Absorption Fine Structure (EXAFS) spectroscopy, as shown in **Figures** **5** and S6 (Supporting Information). The XANES spectra of Ni foil show the edge at ≈8333 eV, characteristic of metallic Ni (Figure 5A).^[^ ^61^ ^]^ In contrast, both Ni/CeO_2_ and Ni‐Pr/CeO_2_ catalysts exhibit a higher energy edge shift and a more intense absorption peak than Ni foil but a less intense peak compared to NiO, indicating a predominant Ni^2+^ oxidation state.^[^ ^10^, ^62^ ^]^ However, the XANES spectrum of Ni‐Pr/CeO_2_, shifted to slightly higher energy than Ni foil and lower energy than Ni/CeO_2_ catalysts, suggesting cationic state Ni^2+δ^ or mixed oxidation state (Ni^2+^ / Ni^3+^) due to the passivation or charge transfer of Ni metal to ceria support.^[^ ^7^, ^62^, ^63^ ^]^ The Fourier transform EXAFS R‐space spectra (Figure 5B) reveal that Ni‐Ni coordination, characteristic of metallic Ni, is absent in both Ni/CeO_2_ and Ni‐Pr/CeO_2_ catalysts. Instead, a distinct Ni‐O peak, consistent with the NiO reference, is observed. The EXAFS fitting results (**Table** **2**) confirm that both Ni‐Ni and Ni‐O coordination numbers in the catalysts match those of NiO nanoparticles, suggesting that Ni exists primarily as dispersed NiO‐like species, rather than forming a strong interaction with the ceria lattice.^[^ ^64^ ^]^ The best fits to the EXAFS data, performed in R‐space using a NiO rocksalt structure model, confirm that the Ni‐O coordination number (CN) is 5.8 ± 0.2 for Ni/CeO_2_ and 5.9 ± 1.6 for Ni‐Pr/CeO_2_, comparable to the reference NiO standard (CN ≈6).^[^ ^65^, ^66^ ^]^ The ratio CN_(Ni‐O)_/CN_(Ni‐Ni)_ in Ni/CeO_2_ (0.49) and Ni‐Pr/CeO_2_ (0.50) is also similar to NiO, confirming that the Ni‐O coordination originates from NiO nanoparticles rather than strong bonding with ceria support. This result revealed that Ni─O bonds are coming from only NiO nanoparticles of both the catalysts and confirmed the interaction of Ni with interfacial O atoms only, not from ceria support, suggesting that Ni exists primarily as dispersed NiO‐like species, rather than forming a strong interaction with the ceria support.^[^ ^25^, ^67^ ^]^ Upon Pr doping, a peak splitting in the Ni─O bond is observed, which may arise due to an increase in positive charge on Ni. This is further corroborated by the slightly higher Ni‐Ni coordination number (11.7 ± 3.1) in Ni‐Pr/CeO_2_ than Ni/CeO_2_ (11.7 ± 0.5).^[^ ^10^ ^]^ The Pr L_3_‐edge XANES spectrum (Figure 5C) of Ni‐Pr/CeO_2_ was compared to a Pr_2_O_3_ reference. A peak at 5970 eV, assigned to Pr^3+^, was observed with a higher intensity than in Pr_2_O_3,_ indicating an increased Pr^3+^ population.^[^ ^53^, ^68^ ^]^ Ce L_3_‐edge XANES spectra of Ni‐Pr/CeO_2_ and Ni/CeO_2_ catalysts exhibit peaks at 5732 and 5738 eV corresponding to Ce^4+^ oxidation state (Figure 5D).^[^ ^69^, ^70^ ^]^ The oxidation state of cerium in CeO_2_, as determined by XANES, remains a subject of debate within the scientific community due to varying interpretations of the 2p → 5d excitation.^[^ ^71^, ^72^ ^]^ The peak at 5732 and 5739 eV corresponds to 2p_3/2_→(4fL)5d and 2p_3/2_→(4f^0^)5d emissions, respectively, where L represents oxygen vacancy or hole in ceria lattice.^[^ ^73^, ^74^ ^]^ However, the hybridization between Ce 4f and O 2p orbitals facilitates electron density transfer from oxygen to cerium, resulting in a mixed oxidation state.^[^ ^75^, ^76^ ^]^ The white line intensity of the CeO_2_ standard is higher than Ni/CeO_2_ and Ni‐Pr/CeO_2_ catalysts (Figure 5D,E) indicating the reduction of Ce^4+^ to Ce^3+^ state.^[^ ^74^ ^]^

**Table 2:** Ni K‐edge fitting results.

Sample	Path	*R* (Å)	*CN*	*σ* ^2^	R‐factor
NiO	Ni‐O	2.06 ± 0.01	6 (fixed)	0.006	0.010
	Ni‐Ni	2.95 ± 0.02	12 (fixed)	0.007	
Ni/CeO_2_	Ni‐O	2.06 ± 0.03	5.8 ± 0.2	0.006	0.008
	Ni‐Ni	2.95 ± 0.02	11.7 ± 0.5	0.006	
Ni‐Pr/CeO_2_	Ni‐O	2.06 ± 0.03	5.9 ± 1.6	0.005	0.014
	Ni‐Ni	2.95 ± 0.02	11.7 ± 3.1	0.003	

Where, R: interatomic distance; *CN*: coordination number; *σ* ^2^: Debye‐Waller factor and R‐factor: goodness of fit.

Further, the best fits of EXAFS data were performed in R‐space for CeO_2_, Ni/CeO_2_, and Ni‐Pr/CeO_2_, and the obtained parameters are shown in **Table** **3**. This result revealed the increment of Ce─O bond length in Ni‐Pr/CeO_2_ after doping of Pr^3+^ ion when compared with Ni/CeO_2_ and CeO_2,_ indicating isolated Pr^3+^ ions over ceria support in Ni‐Pr/CeO_2_ catalyst.^[^ ^1^, ^53^, ^77^, ^78^ ^]^ Also, a decrease in Ce‐O coordination number from 6.8 ± 2.9 (Ni/CeO_2_) to 4.6 ± 2.0 (Ni‐Pr/CeO_2_), confirming increased oxygen defect formation upon Pr^3+^ incorporation.

**Table 3:** Ce L_3_‐edge fitting results.

Sample	Path	*R* (Å)	*CN*	*σ* ^2^	R‐factor
CeO_2_	Ce‐O	2.30 ± 0.03	8 (fixed)	0.016	0.014
Ni/CeO_2_	Ce‐O	2.29 ± 0.04	6.8 ± 2.9	0.011	0.018
Ni‐Pr/CeO_2_	Ce‐O	2.33 ± 0.04	4.6 ± 2.0	0.005	0.012

**Figure 6:**
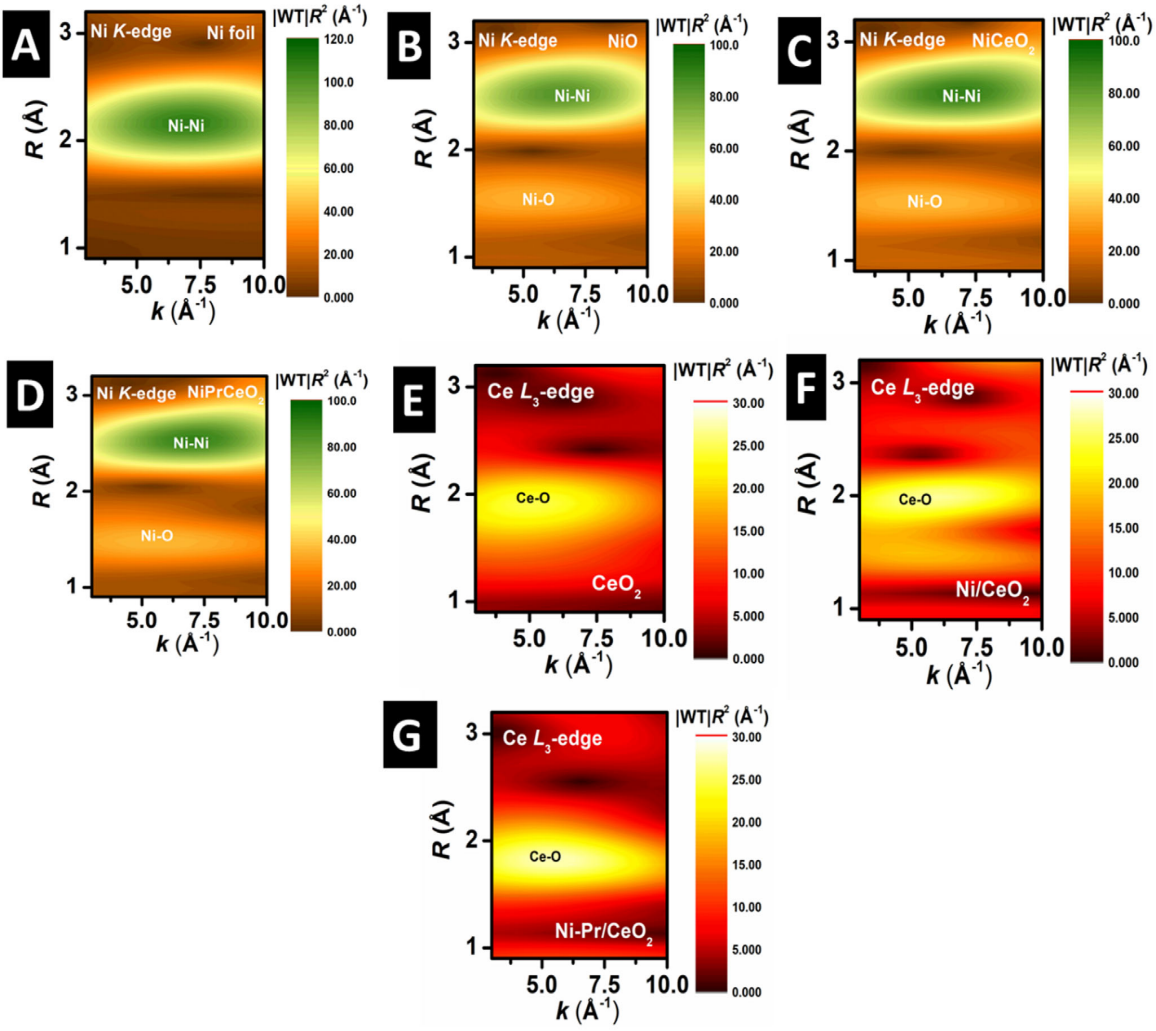
Morlet wavelet transforms of NiO A), Ni foil B), Ni/CeO_2_ C), and Ni‐Pr/CeO_2_ D) for Ni K‐edge and CeO_2_ E), Ni/CeO_2_ F) and Ni‐Pr/CeO_2_ G) for Ce L_3_‐edge.

To further distinguish overlapping scattering contributions, wavelet transform (WT) analysis of the k^3^‐weighted EXAFS spectra was performed as shown in **Figure** **6**. In NiO, Ni/CeO_2_, and Ni‐Pr/CeO_2_, two lobes corresponding to Ni‐Ni and Ni‐O shells were observed (Figure 6A–D). Compared to Ni foil, the Ni‐Ni feature is significantly weaker in Ni/CeO_2_, and Ni‐Pr/CeO_2_, indicating a high dispersion of Ni species rather than bulk Ni clusters. Meanwhile, the Ni‐O signal is more pronounced, suggesting that Ni is predominantly coordinated with oxygen within the catalysts. The Ce L_3_‐edge WT analysis further refined our understanding of the structural changes induced by Ni and Pr doping in CeO_2,_ as shown in Figure 6E–G. The WT spectra of CeO_2_, Ni/CeO_2_, and Ni‐Pr/CeO_2_ revealed systematic shifts in the intensity and position of scattering features, corresponding to variations in Ce‐O and Ce‐Ce interactions.^[^ ^77^ ^]^ In pure CeO_2_, the dominant Ce‐O scattering feature remained well‐defined, but with Ni incorporation (Ni/CeO_2_), a broadening and shift toward lower k‐space values indicated increased disorder and a higher fraction of Ce^3+^ species.^[^ ^79^, ^80^ ^]^ This trend became even more pronounced in Ni‐Pr/CeO_2_, where the Ce‐O contribution weakened, confirming an increase in oxygen vacancies. The distinct WT signature of Ni‐Pr/CeO_2_ reflected a more disrupted local environment, consistent with more defective and catalytically active ceria support.

**Figure 7:**
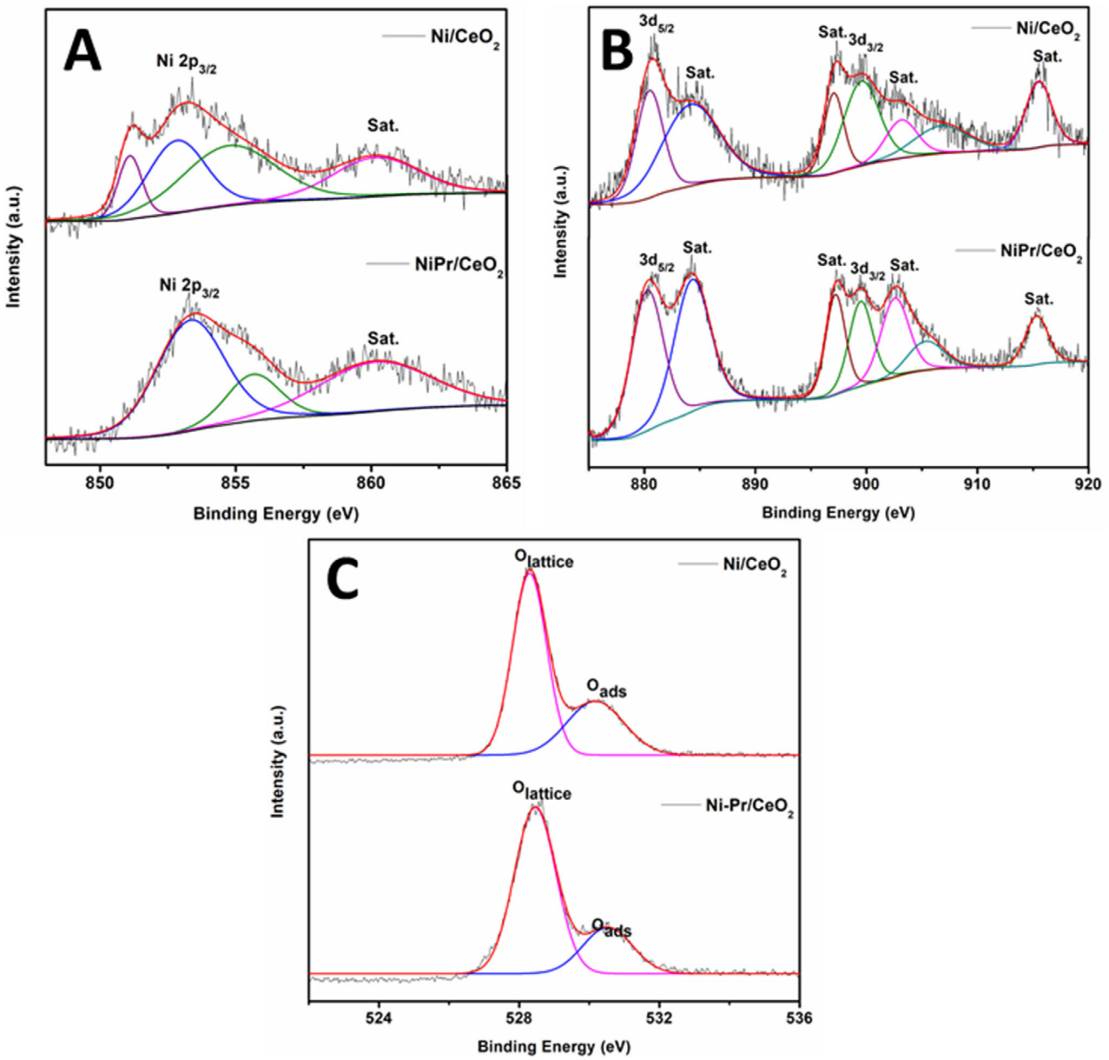
XPS spectra of A) Ni 2p, B) Ce 3d, and C) O 1s for Ni/CeO_2_ and Ni‐Pr/CeO_2_ catalysts.

X‐ray photoelectron spectroscopy (XPS) was performed on reduced Ni‐Pr/CeO_2_ and Ni/CeO_2_ catalysts for Ni 2p, Ce 3d, and O 1s spectra, as shown in **Figure** **7**. The deconvolution of all obtained spectra was done considering 284.8 eV as a reference. The Ni 2p spectra of Ni‐Pr/CeO_2_ displayed a peak for metallic Ni(0) at 853.16 eV and for Ni(+2) at 854.16 and 860.08 eV as shown in Figure 7A, whereas in Ni/CeO_2_, the peaks at 853.35 eV for metallic Ni(0) and 851.1, 855.02 and 860.1 eV corresponds to Ni^2+^.^[^ ^7^, ^81^, ^82^ ^]^ The slight shift toward lower binding energy in the case of Ni‐Pr/CeO_2_ corresponds to a shift in electron density after doping of Pr^3+^ over ceria. This result also suggests that Pr doping enhances electron density at the Ni site in comparison to the undoped Ni site, which helps to enhance the dissociation of H_2_ and CO_2_ on the catalyst surface.^[^ ^7^, ^10^, ^32^ ^]^ The ratio between Ni(0)/Ni(+2) was also analyzed, which revealed that in Ni‐Pr/CeO_2_, more metallic content present than Ni/CeO_2_ (**Table** **4**). Nevertheless, the oxidation may occur due to fast surface oxidation of Ni in between recovery of the sample and analysis time.

**Table 4:** Surface quantification for catalysts using XPS analysis.

Sample	Ni^0^/Ni^2+^	Ce^3+^/Ce^3+^+Ce^4+^	Ce^3+^/Ce^4+^	O_ads_/(O_ads_+O_latt_)
Ni/CeO_2_	0.75	71.06	2.02	24.91
Ni‐Pr/CeO_2_	3.26	76.41	4.98	33.09

The Ce 3d spectra were also performed which showed peaks at 880.46, 884.16, 899.54, 903.14 eV for Ce^3+^ and at 897.05, 906.70 eV for Ce^4+^ in Ni‐Pr/CeO_2_ catalysts whereas at 880.27, 884.30, 899.49 and 902.56 eV for Ce^3+^ and at 897.21, 905.34 and 915.33 eV for Ce^4+^ cations (Figure 7B).^[^ ^29^, ^83^ ^]^ Furthermore, to analyze the H‐spillover effect, the Ce^3+^/Ce^3+^+Ce^4+^ ratio was calculated using the integral area under the peaks, presented in Table 4. It was reported that for Ni‐Pr/CeO_2_ catalyst showed a higher value than Ni/CeO_2,_ suggesting that Pr^3+^ doping enhances the interfacial sites as well as surface‐absorbed oxygen.^[^ ^84^ ^]^ This may be caused by the H‐spillover effect where Ce^3+^ ions increase which is also confirmed with H_2_‐TPD results. Additionally, O 1s spectra indicate the major peak at ≈528.6 and ≈530.7 eV, corresponding to lattice oxygen (O_latt_) and surface‐adsorbed oxygen (O_ads_) for both Ni/CeO_2_ and Ni‐Pr/CeO_2_ catalysts (Figure 7C).^[^ ^85^, ^86^ ^]^ The O_ads_/(O_ads_+O_latt_) was calculated using the integral area to analyze the oxygen vacancies at the surface of the catalyst, as mentioned in Table 4.

The results revealed that doping of Pr^3+^ enhances the oxygen vacancies represented by a higher ratio for O_ads_/(O_ads_+O_latt_). Overall, these findings suggest that the doping of Pr^3+^ promotes the formation of oxygen vacancies by forming Ce^3+^ ions in the Ni‐Pr/CeO_2_ catalyst. The higher Ce^3+^ fraction in Ni‐Pr/CeO_2_(76.41%) compared to Ni/CeO_2_ (71.06%) is consistent with EXAFS data, reinforcing the conclusion that Pr doping promotes defect formation in ceria, leading to increased oxygen vacancy concentrations.^[^ ^27^, ^36^, ^53^ ^]^

### 2.2 Catalytic Results

**Figure 8:**
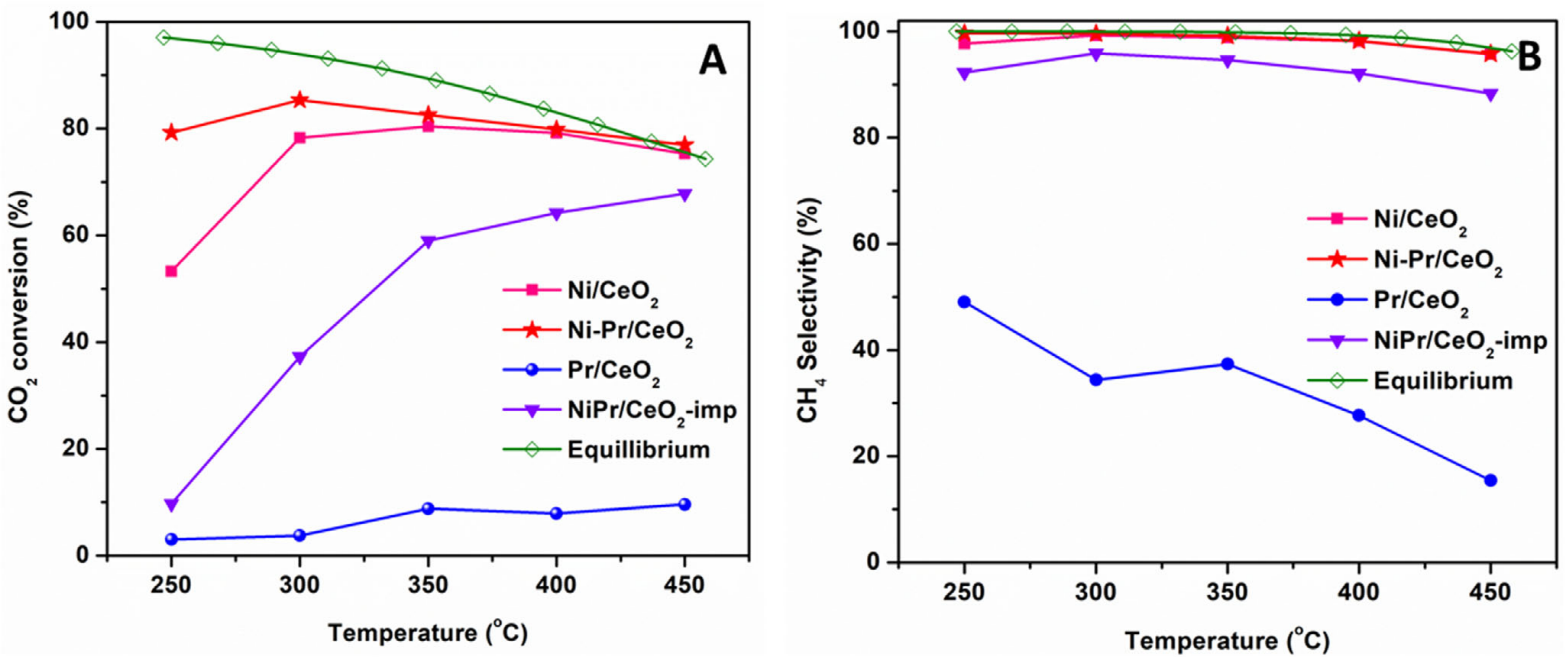
Methanation catalytic test results for all catalysts A) conversion and B) selectivity.

All characterized catalysts were further tested for CO_2_ conversion to methane at 25000 h^−1^ GHSV with varying temperatures from 250–450 °C as shown in **Figure** **8**. In Pr/CeO_2_, the conversion as well as selectivity was significantly low owing to the formation of more CO than CH_4_ as a product (Figure 8A,B). This result was consistent even at higher temperatures, i.e., 350–450 °C. The desorption of CO was maybe easy due to weaker binding with Pr sites in the Pr/CeO_2_ catalyst. It was reported that the higher amount of Pr over ceria affects both the conversion and selectivity of methane.^[^ ^35^, ^38^, ^53^ ^]^ Furthermore, the atomically dispersed Ni‐Pr/CeO_2_ catalyst showed highest catalytic activity over undoped Ni/CeO_2_. The doping of Pr^3+^ over ceria enhances the catalytic activity drastically from 53 to 80%, even at 250 °C temperature. Even at 300 °C, the Ni‐Pr/CeO_2_ catalyst showed excellent 87% conversion with ≈100% selectivity toward methane product. When the catalytic temperature increased from 250–450 °C, the conversion increased 53%, 87%, 82%, 80%, and 77% at 250, 300, 350, 400 and 450 °C temperatures, respectively. This result revealed that the doping of Pr^3+^ plays an important role by enhancing the oxygen vacancies and hydrogen spillover at low reaction temperatures, as discussed above.^[^ ^33^, ^53^ ^]^ When the catalytic activity was compared with an impregnated NiPr/CeO_2_‐imp catalyst, only 37% conversion was obtained at 300 °C temperature with a decrease in selectivity. This result is in good agreement with other results presented in this study, where Pr/CeO_2_ showed activity toward CO formation, and Ni/CeO_2_ showed moderate activity. The trend of catalytic activity was as follows: Ni‐Pr/CeO_2_ > Ni/CeO_2_ > NiPr/CeO_2_ > Pr/CeO_2_.

The catalytic performance of Ni‐Pr/CeO_2_ catalyst is superior over previously reported Pr, Ni, and CeO_2_‐based catalysts, as shown in Table S3 (Supporting Information). Tsiotsias et al. reported Pr‐based three catalysts (10 mol% Pr loading) using different synthesis methods at 350 °C, the maximum conversion was 77% with 99% selectivity for methane production at 25 000 ml g h^−1^ WHSV, whereas at 300 °C the conversion was only 46%, 33%, 71%, respectively for Ni/CSG, Ni/PC and Ni/MPC (Entrys 1–3).^[^ ^39^ ^]^ Another study utilized the citrate sol‐gel method with Pr loadings ranging from 5–50 atomic % along with Ni and ceria, achieving only 45–21% conversion at 300 °C (Entrys 4–7).^[^ ^38^ ^]^ The findings indicated that increasing the Pr content led to a decline in both conversion and selectivity. Siakavelas et al. prepared a catalyst using the microwave method, followed by the sol‐gel method, i.e., Ni/Pr‐Ce, which showed only 24% conversion with 25 000 mL g h^−1^ weight basis GHSV (Entry 8).^[^ ^33^ ^]^ In another report, they utilized multimetallic catalytic system for methanation, i.e., Ni/La‐Pr‐CeO_2_ where 10% Ni, 5% La_2_O_3_ and 10% Pr_2_O_3_‐CeO_2_ was prepared using microwave sol‐gel method and then the wet‐impregnation method at 25 000 mL g_cat_ h^−1^ which displayed 21% conversion at 300 °C whereas 55% conversion at 350 °C temperature with 100% selectivity (Entry 9).^[^ ^87^ ^]^ Rodriguez et al. reported Ru‐based Pr‐CeO_2_ catalyst using the mechanochemical method followed by the incipient wetness method, which showed 81% conversion at very low GHSV, i.e., 9000 h^−1^ and 500 °C temperature (Entry 10).^[^ ^35^ ^]^ Based on these reports, we can conclude that the synthesized atomically dispersed Ni‐Pr/CeO_2_ catalyst in this study showed excellent and superior catalytic activity even at lower reaction temperatures (250–300 °C) at 25 000 h^−1^ GHSV with 100% selectivity toward methane. These results also support that uniform dispersion of Pr^3+^ ion over ceria support along with Ni nanoparticles drastically enhanced the catalytic activity without compromising the selectivity toward methane production. Also, the synthesis processes of the above‐mentioned catalysts involved a multistep process with precious metals like Ru, Pd and high reduction temperature. Based on this, our results indicate superior catalytic performance with optimal reaction conditions and low reaction and reduction temperature with low Pr metal loading, i.e., 8 wt.% with 25 000 h^−1^ GHSV with excellent catalytic performance. To the best of our knowledge, the isolated atom Pr^3+^ over ceria support and NiO decorated catalyst is superior to other reported Pr, Ni, and ceria‐based catalysts for CO_2_ methanation catalysis at low reaction temperatures.

**Figure 9:**
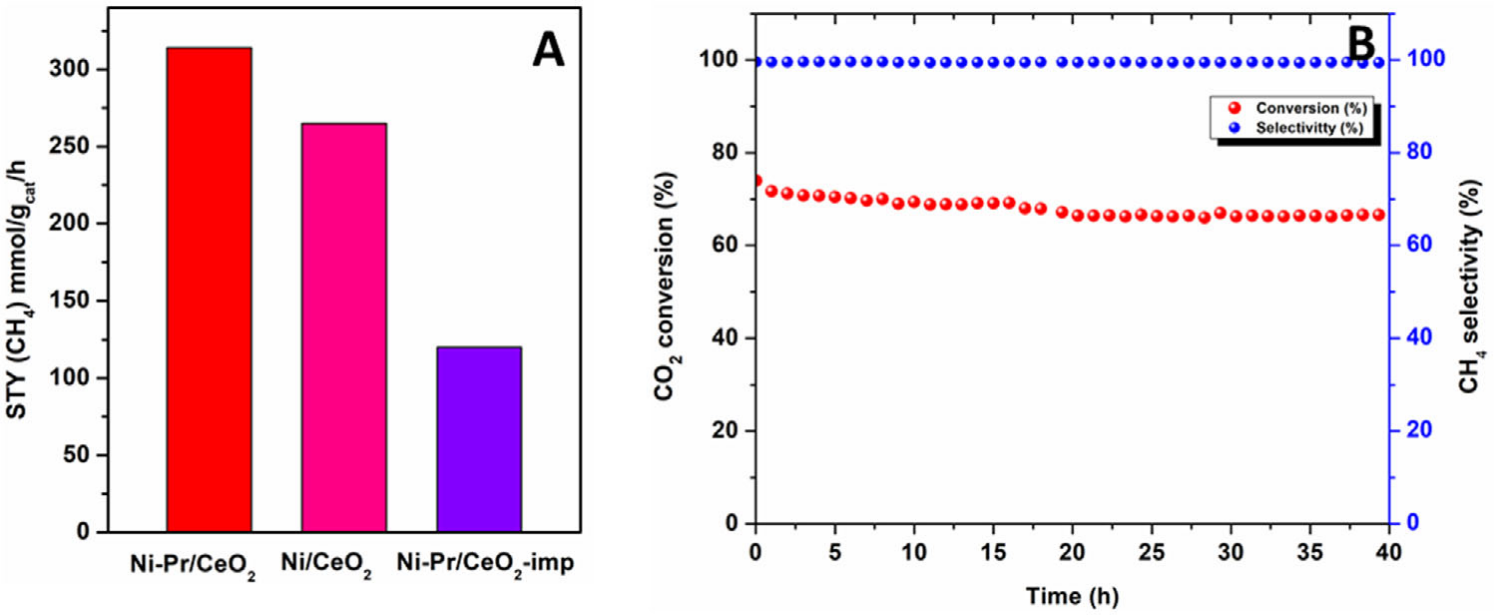
A) Space‐time yield (STY) of methane at 300 °C and B) long‐term CO_2_ conversion of Ni‐Pr/CeO_2_ catalyst at 275 °C (40 h).

The space‐time yield (STY) for methane was also calculated at 300 °C temperature at 100 mL min^−1^ flow rate and 25 000 h^−1^ GHSV, as shown in **Figure** **9**A. The obtained result showed superior STY for Ni‐Pr/CeO_2_ atomically dispersed catalyst than impregnated NiPr/CeO_2_ and Ni/CeO_2_ catalysts, i.e., 314 mmol g_cat_^−1^ h^−1^. The long‐run test was performed to analyze the robustness of the best catalyst Ni‐Pr/CeO_2_. In this regard, the catalyst was taken and reduced at 400 °C and then kept for 40 h at 275 °C under CO_2_:H_2_ = 1:4 ratio, as shown in Figure 9. The catalyst showed no significant change in catalytic activity and selectivity toward methane. This result showed high thermal stability and reusability of the catalyst, which could pave the path for bulk‐scale application.

After 40 h of catalytic runs, the HRTEM and PXRD analysis was analyzed. No significant changes in the catalytic system were observed. In summary, it can be mentioned that the Pr‐doped Ni/CeO_2_ catalyst showed superior catalytic activity over Ni/CeO_2_ and NiPr/CeO_2_‐imp catalysts. The reason behind such excellent catalytic performance was estimated as H‐spillover and high oxygen vacancy with a uniform dispersion of Pr over ceria support. Also, each Pr^3+^ ions act as an active site for the catalytic conversion of CO_2_ to methane.

### 2.3 Structure‐Performance Relationship

Based on comprehensive characterization, catalytic performance, and insights from previous literature, the structure‐performance relationship of the optimized Ni‐Pr/CeO_2_ catalyst for CO_2_ methanation was established. The atomically dispersed Ni‐Pr/CeO_2_ catalyst demonstrated significantly higher catalytic activity than Ni/CeO_2_ and NiPr/CeO_2_‐imp catalysts. This enhanced performance can be attributed to key factors such as improved reducibility, increased basicity, enhanced metal dispersion, stronger metal‐support interactions, the presence of oxygen vacancies, and optimized dopant concentration.

The isolated Pr^3+^ ions on the ceria support while simultaneously strengthening the interaction between NiO and CeO_2_ in the Ni‐Pr/CeO_2_ system. This observation aligns with H_2_‐TPR results, where Pr doping lowered the reduction temperature (Figure 2A), indicating improved reducibility. Besides, CO_2_‐TPD analysis revealed a higher concentration of basic sites within the 300–500 °C range, along with an overall increase in basicity compared to undoped Ni/CeO_2_ (Figure 2B). These basic sites play a crucial role in enhancing catalytic efficiency at lower reaction temperatures by accelerating the conversion of formate and carbonate intermediates into methane, as evidenced in this study.^[^ ^84^ ^]^

The uniform dispersion of Pr^3+^ on the ceria support was confirmed through STEM‐EDS analysis, revealing a concentration of approximately ≈7 wt.% (Figure 4), which aligns well with the ICP‐OES results (Table S1, Supporting Information). This atomically dispersed Pr^3+^ plays a crucial role in enhancing catalytic performance.

To further investigate the role of Ni species, the Pr/CeO_2_ catalyst was tested for methane formation. Its poor catalytic performance confirmed that the presence of Ni, along with Pr and Ce, is essential for achieving superior catalytic activity. A review of the literature indicates that Ni nanoparticles in the range of 10–25 nm, when supported on CeO_2_, exhibit highly effective CO_2_ methanation activity.^[^ ^39^, ^52^, ^88^ ^]^ This finding aligns with the crystallite size obtained from PXRD analysis in this study (Table S2, Supporting Information).

Furthermore, the number of interfacial sites and the amount of metallic Ni were quantified through XPS and XAS analyses, revealing higher values in Ni‐Pr/CeO_2_ compared to Ni/CeO_2_ (Table 4). This confirms that Pr^3+^ doping enhances the interfacial sites while maintaining metallic Ni. The balance between interfacial sites Ni‐O‐Ce and metallic Ni needs to be maintained because Ni‐O‐Ce plays a key role in CO_2_ activation while Ni(0) activates the hydrogen for reaction.^[^ ^89^, ^90^, ^91^ ^]^

Additionally, H_2_ dissociation and spillover at metal‐support interfacial sites were confirmed by H_2_‐TPD, H_2_‐TPR, and XPS analyses, which demonstrated their role in promoting CO_2_ conversion and methane selectivity.^[^ ^47^, ^84^ ^]^ The lattice defects and oxygen mobility within the CeO_2_ structure significantly influence CO_2_ adsorption, further enhancing catalytic performance.^[^ ^92^, ^93^, ^94^ ^]^ In the case of Ni‐Pr/CeO_2_, XPS analysis revealed that the Ce^3+^/Ce^4+^ ratio was more than twice as high as in Ni/CeO_2_ (Table 4). This defect formation in the ceria lattice is attributed to the size difference between Pr^3+^ (1.27 Å) and Ce^4+^ (0.97 Å), which leads to structural distortions in the ceria fluorite lattice as Pr^3+^ replaces Ce^4+^. PXRD analysis further supports this structural modification, as evidenced by shifts in the (111) plane peak of ceria (Figure 1B). These findings are consistent with WT results at the Ce L_3_‐edge, which indicate changes in Ce–O bonding upon Pr^3+^ doping in CeO_2_ (Figure 6). Ni K‐edge WT revealed that a high dispersion of Ni species rather than the formation of bulk Ni clusters as well as the Ni species is primarily coordinated with oxygen within the catalyst.

Ahn et al. previously reported that Pr^3+^ doping induces structural distortions in ceria and generates oxygen vacancies by replacing Ce^4+^ within the crystal lattice.^[^ ^53^ ^]^ In this study, Pr^3+^ incorporation was found to create local lattice distortions near oxygen vacancies due to the size mismatch between Pr^3+^ and Ce^4+^, as well as differences in their bond lengths with oxygen. As a result, oxygen mobility within the lattice was enhanced. This observation is well supported by XPS and EXAFS analyses, which confirmed an increase in oxygen vacancies in Ni‐Pr/CeO_2_ catalysts.

In summary, atomically dispersed Pr^3+^ in the Ni/CeO_2_ catalyst improves oxygen vacancy concentration, promotes Ni dispersion, and enhances Ni–O–Ce interfacial sites. Based on previous reports, it can be concluded that Pr^3+^ doping accelerates the transformation of formate and carbonate intermediates, while Ni species facilitate hydrogen spillover and CO_2_ activation.^[^ ^10^, ^38^, ^89^ ^]^ This study demonstrates that the substitution of CeO_2_ with a small amount of Pr is an effective strategy for developing highly active Ni‐based CO_2_ methanation catalysts.

## 3 Conclusion

Pr^3+^ doped CeO_2_ and decorated with NiO nanoparticles were prepared via the co‐precipitation method and utilized for excellent catalytic conversion of CO_2_ to methane. XAS, STEM‐EDS analysis confirmed the presence of atomically dispersed and isolated Pr^3+^ ions as isolated atoms over ceria support. The advantage of isolated ions was studied by comparing the activity with undoped Ni/CeO_2_ and impregnated Ni‐Pr/CeO_2_ catalysts where uniformly dispersed Ni‐Pr/CeO_2_ showed superior catalytic activity with 87% conversion and 100% selectivity at low reaction temperature. The results suggest that Pr^3+^ doping promotes interfacial sites, oxygen vacancies, and hydrogen spillover to enhance the reaction of methanation.

## Conflict of Interest

The authors declare no conflict of interest.

## Supporting information



Supporting Information

## Data Availability

The data that support the findings of this study are available on request from the corresponding author. The data are not publicly available due to privacy or ethical restrictions.

## References

[1] N. Choudhary , S. Jiang , H. Pham , G. Kedarnath , A. Datye , J. T. Miller , A. K. Tyagi , M. M. Shaikh , Appl. Catal. B Environ. 2024, 344, 123627.

[2] M. Fan , J. D. Jimenez , S. N. Shirodkar , J. Wu , S. Chen , L. Song , M. M. Royko , J. Zhang , H. Guo , J. Cui , K. Zuo , W. Wang , C. Zhang , F. Yuan , R. Vajtai , J. Qian , J. Yang , B. I. Yakobson , J. M. Tour , J. Lauterbach , D. Sun , P. M. Ajayan , ACS Catal. 2019, 9, 10077.

[3] N. Choudhary , K. Nabeela , N. Mate , S. M. Mobin , RSC Sustain. 2024, 2, 1179.

[4] X. Shi , Y. Huang , Y. Bo , D. Duan , Z. Wang , J. Cao , G. Zhu , W. Ho , L. Wang , T. Huang , Y. Xiong , Angew. Chem. 2022, 134, 202203063. 10.1002/anie.202203063 35475563

[5] M. Liu , R. Zou , C. Liu , Appl. Catal. B Environ. Energy 2025, 360, 124549.

[6] M. Zhang , Y. Mao , X. Bao , P. Wang , Y. Liu , Z. Zheng , H. Cheng , Y. Dai , Z. Wang , B. Huang , ACS Catal. 2024, 14, 5275.

[7] T. Zhang , P. Zheng , F. Gu , W. Xu , W. Chen , T. Zhu , Y.‐F. Han , G. Xu , Z. Zhong , F. Su , Appl. Catal. B Environ. 2023, 323, 122190.

[8] S. Chen , Z. Zhang , W. Jiang , S. Zhang , J. Zhu , L. Wang , H. Ou , S. Zaman , L. Tan , P. Zhu , E. Zhang , P. Jiang , Y. Su , D. Wang , Y. Li , J. Am. Chem. Soc. 2022, 144, 12807. 35786905 10.1021/jacs.2c03875

[9] J. Wang , E. Kim , D. P. Kumar , A. P. Rangappa , Y. Kim , Y. Zhang , T. K. Kim , Angew. Chem. 2022, 134, 202113044. 10.1002/anie.202113044 34750936

[10] Z. Zhang , Z. Yu , K. Feng , B. Yan , Appl. Catal. B Environ. 2022, 317, 121800.

[11] T. Yarbaş , N. Ayas , Int. J. Hydrog. Energy 2024, 49, 1134.

[12] H. Jiang , L. Wang , H. Kaneko , R. Gu , G. Su , L. Li , J. Zhang , H. Song , F. Zhu , A. Yamaguchi , J. Xu , F. Liu , M. Miyauchi , W. Ding , M. Zhong , Nat. Catal. 2023, 6, 519.

[13] J. Kumar Prabhakar , P. A. Apte , G. Deo , Chem. Eng. J. 2023, 471, 144252.

[14] H. Kang , J. Ma , S. Perathoner , W. Chu , G. Centi , Y. Liu , Chem. Soc. Rev. 2023, 52, 3627. 37158259 10.1039/d2cs00214k

[15] O. E. Medina , A. A. Amell , D. López , A. Santamaría , Renew. Sustain. Energy Rev. 2025, 207, 114926.

[16] L. H. Vieira , L. F. Rasteiro , C. S. Santana , G. L. Catuzo , A. H. M. da Silva , J. M. Assaf , E. M. Assaf , ChemCatChem 2023, 15, 202300493.

[17] M. Younas , L. L. Kong , M. J. K. Bashir , H. Nadeem , A. Shehzad , S. Sethupathi , Energy Fuels 2016, 30, 8815.

[18] J. Li , K. Li , Z. Li , C. Wang , Y. Liang , Y. Pang , J. Ma , F. Wang , P. Ning , H. He , Nat. Commun. 2024, 15, 3874. 38719826 10.1038/s41467-024-47836-x PMC11078991

[19] P. Gao , S. Tang , X. Han , Z. Hao , J. Chen , Y. Pan , Z. Zhang , H. Zhang , X. Zi , L. Chen , M. Li , X. Ma , Chem. Eng. J. 2024, 498, 155784.

[20] R. Qiu , W. Wang , Z. Wang , H. Wang , Catal. Sci. Technol. 2023, 13, 2566.

[21] S. Bhat , M. Sepúlveda‐Pagán , J. Borrero‐Negrón , J. E. Meléndez‐Gil , E. Nikolla , Y. J. Pagán‐Torres , Catal. Sci. Technol. 2024, 14, 3364.

[22] W. Jiang , H. Loh , B. Q. L. Low , H. Zhu , J. Low , J. Z. X. Heng , K. Y. Tang , Z. Li , X. J. Loh , E. Ye , Y. Xiong , Appl. Catal. B Environ. 2023, 321, 122079.

[23] N. H. M. Dostagir , R. Rattanawan , M. Gao , J. Ota , J. Hasegawa , K. Asakura , A. Fukouka , A. Shrotri , ACS Catal. 2021, 11, 9450.

[24] W. L. Vrijburg , J. W. A. van Helden , A. Parastaev , E. Groeneveld , E. A. Pidko , E. J. M. Hensen , Catal. Sci. Technol. 2019, 9, 5001.

[25] N. Rui , X. Zhang , F. Zhang , Z. Liu , X. Cao , Z. Xie , R. Zou , S. D. Senanayake , Y. Yang , J. A. Rodriguez , C.‐J. Liu , Appl. Catal. B Environ. 2021, 282, 119581.

[26] R. Tang , N. Ullah , Y. Hui , X. Li , Z. Li , Mol. Catal. 2021, 508, 111602.

[27] W. Yang , K. Chang , M. Yang , X. Yan , S. Yang , Y. Liu , G. Wang , F. Xia , H. Wang , Q. Zhang , Chem. Eng. J. 2024, 499, 156493.

[28] Z. Xiao , Y. Li , F. Hou , C. Wu , L. Pan , J. Zou , L. Wang , X. Zhang , G. Liu , G. Li , Appl. Catal. B Environ. 2019, 258, 117940.

[29] X. Xu , L. Liu , Y. Tong , X. Fang , J. Xu , D. Jiang , X. Wang , ACS Catal. 2021, 11, 5762.

[30] C. Sun , P. Beaunier , V. La Parola , L. F. Liotta , P. D. Costa , ACS Appl. Nano Mater. 2020, 3, 12355.

[31] F. Liu , Y. S. Park , D. Diercks , P. Kazempoor , C. Duan , ACS Appl. Mater. Interfaces 2022, 14, 13295. 35262347 10.1021/acsami.1c23881

[32] T. Zhang , W. Wang , F. Gu , W. Xu , J. Zhang , Z. Li , T. Zhu , G. Xu , Z. Zhong , F. Su , Appl. Catal. B Environ. 2022, 312, 121385.

[33] G. I. Siakavelas , N. D. Charisiou , S. AlKhoori , A. A. AlKhoori , V. Sebastian , S. J. Hinder , M. A. Baker , I. V. Yentekakis , K. Polychronopoulou , M. A. Goula , Appl. Catal. B Environ. 2021, 282, 119562.

[34] P. Parsai , N. Choudhary , R. Sahu , S. M. Mobin , Chem. – Asian J. 2024, 10.1002/asia.202401395. 39714379

[35] S. L. Rodríguez , A. Davó‐Quiñonero , J. Juan‐Juan , E. Bailón‐García , D. Lozano‐Castelló , A. Bueno‐López , J. Phys. Chem. C 2021, 125, 12038. 10.1021/acs.jpcc.1c03539 PMC8494384 34630817

[36] S. Ballauri , E. Sartoretti , M. Hu , C. D'Agostino , Z. Ge , L. Wu , C. Novara , F. Giorgis , M. Piumetti , D. Fino , N. Russo , S. Bensaid , Appl. Catal. B Environ. 2023, 320, 121898.

[37] K. Chen , W. Li , X. Li , A. T. Ogunbiyi , L. Yuan , ACS Appl. Nano Mater 2021, 4, 5404.

[38] A. I. Tsiotsias , N. D. Charisiou , A. AlKhoori , S. Gaber , V. Stolojan , V. Sebastian , B. van der Linden , A. Bansode , S. J. Hinder , M. A. Baker , K. Polychronopoulou , M. A. Goula , J. Energy Chem. 2022, 71, 547.

[39] A. I. Tsiotsias , N. D. Charisiou , E. Harkou , S. Hafeez , G. Manos , A. Constantinou , A. G. S. Hussien , A. A. Dabbawala , V. Sebastian , S. J. Hinder , M. A. Baker , K. Polychronopoulou , M. A. Goula , Appl. Catal. B Environ. 2022, 318, 121836.

[40] N. Guillén‐Hurtado , J. Giménez‐Mañogil , J. C. Martínez‐Munuera , A. Bueno‐López , A. García‐García , Appl. Catal. Gen. 2020, 590, 117339.

[41] N. Choudhary , V. Kumar , S. M. Mobin , ChemistrySelect 2022, 7, 202202501.

[42] L. P. Martin , D. Nagle , M. Rosen , Mater. Sci. Eng. A 1998, 246, 151.

[43] C. Lyu , X. Zhou , X. Lu , Y. Zhang , C. Li , Q. Zhou , Z. Sun , G. Chen , Geofluids 2021, 2021, 8898142.

[44] G. Gao , J. Remón , Z. Jiang , L. Yao , C. Hu , Appl. Catal. B Environ. 2022, 309, 121260.

[45] V. G. Baldovino‐Medrano , V. Niño‐Celis , R. Isaacs Giraldo , J. Chem. Eng. Data 2023, 68, 2512.

[46] P. Mierczynski , A. Mierczynska , R. Ciesielski , M. Mosinska , M. Nowosielska , A. Czylkowska , W. Maniukiewicz , M. I. Szynkowska , K. Vasilev , Catalysts 2018, 8, 380.

[47] Y. Guo , S. Mei , K. Yuan , D.‐J. Wang , H.‐C. Liu , C.‐H. Yan , Y.‐W. Zhang , ACS Catal. 2018, 8, 6203.

[48] C. Italiano , J. Llorca , L. Pino , M. Ferraro , V. Antonucci , A. Vita , Appl. Catal. B Environ. 2020, 264, 118494.

[49] Z. Ni , X. Djitcheu , X. Gao , J. Wang , H. Liu , Q. Zhang , Sci. Rep. 2022, 12, 5344. 35351943 10.1038/s41598-022-09291-w PMC8964754

[50] L. Xu , H. Song , L. Chou , Int. J. Hydrog. Energy 2012, 37, 18001.

[51] G. Zhou , H. Liu , K. Cui , A. Jia , G. Hu , Z. Jiao , Y. Liu , X. Zhang , Appl. Surf. Sci. 2016, 383, 248.

[52] Y. Du , C. Qin , Y. Xu , D. Xu , J. Bai , G. Ma , M. Ding , Chem. Eng. J. 2021, 418, 129402.

[53] K. Ahn , D. S. Yoo , D. H. Prasad , H.‐W. Lee , Y.‐C. Chung , J.‐H. Lee , Chem. Mater. 2012, 24, 4261.

[54] R.‐P. Ye , Q. Li , W. Gong , T. Wang , J. J. Razink , L. Lin , Y.‐Y. Qin , Z. Zhou , H. Adidharma , J. Tang , A. G. Russell , M. Fan , Y.‐G. Yao , Appl. Catal. B Environ. 2020, 268, 118474.

[55] M. Li , H. Amari , A. C. van Veen , Appl. Catal. B Environ. 2018, 239, 27.

[56] X. Li , D. Li , H. Tian , L. Zeng , Z.‐J. Zhao , J. Gong , Appl. Catal. B Environ. 2017, 202, 683.

[57] H. Muroyama , Y. Tsuda , T. Asakoshi , H. Masitah , T. Okanishi , T. Matsui , K. Eguchi , J. Catal. 2016, 343, 178.

[58] M. Zhu , P. Tian , X. Cao , J. Chen , T. Pu , B. Shi , J. Xu , J. Moon , Z. Wu , Y.‐F. Han , Appl. Catal. B Environ. 2021, 282, 119561.

[59] R. Mi , D. Li , Z. Hu , R. T. Yang , ACS Catal. 2021, 11, 7876.

[60] T. G. Novak , A. E. Herzog , M. R. Buck , R. J. Spears , K. Sendgikoski , R. H. DeBlock , T. H. Brintlinger , P. A. DeSario , D. R. Rolison , Sci. Adv. 2024, 10, adr9120. 10.1126/sciadv.adr9120 PMC11578172 39565851

[61] S.‐Y. Lee , H.‐J. Oh , M. Kim , H.‐S. Cho , Y.‐K. Lee , Appl. Catal. B Environ. 2023, 324, 122269.

[62] W. Liu , X. Li , Y. Hao , D. Xiong , H. Shan , J. Wang , W. Xiao , H. Yang , H. Yang , L. Kou , Z. Tian , L. Shao , C. Zhang , Adv. Funct. Mater. 2021, 31, 2008301.

[63] G. Fang , Q. Wang , J. Zhou , Y. Lei , Z. Chen , Z. Wang , A. Pan , S. Liang , ACS Nano 2019, 13, 5635. 31022345 10.1021/acsnano.9b00816

[64] M. Filez , E. A. Redekop , H. Poelman , V. V. Galvita , G. B. Marin , Anal. Chem. 2015, 87, 3520. 25704379 10.1021/acs.analchem.5b00109

[65] J. Li , Y. Li , P. K. Routh , E. Makagon , I. Lubomirsky , A. I. Frenkel , J. Synchrotron Radiat. 2021, 28, 1511. 34475298 10.1107/S1600577521007025

[66] N. Prinz , L. Schwensow , S. Wendholt , A. Jentys , M. Bauer , W. Kleist , M. Zobel , Nanoscale 2020, 12, 15800. 32691790 10.1039/d0nr01750g

[67] X. Ao , Y. Kong , S. Zhao , Z. Chen , Y. Li , X. Liao , B. Tian , Angew. Chem., Int. Ed. 2025, 64, 202415036. 10.1002/anie.202415036 39305143

[68] H. Asakura , T. Shishido , S. Fuchi , K. Teramura , T. Tanaka , J. Phys. Chem. C 2014, 118, 20881.

[69] P. A. Kumar , Y. E. Jeong , S. Gautam , H. P. Ha , K. J. Lee , K. H. Chae , Chem. Eng. J. 2015, 275, 142.

[70] W. Li , S. Yamada , T. Hashimoto , T. Okumura , R. Hayakawa , K. Nitta , O. Sekizawa , H. Suga , T. Uruga , Y. Ichinohe , T. Sato , Y. Toyama , H. Noda , T. Isobe , S. Takatori , T. Hiraki , H. Tatsuno , N. Kominato , M. Ito , Y. Sakai , H. Omamiuda , A. Yamaguchi , T. Yomogida , H. Miura , M. Nagasawa , S. Okada , Y. Takahashi , Anal. Chim. Acta. 2023, 1240, 340755. 36641142 10.1016/j.aca.2022.340755

[71] K. O. Kvashnina , Chem. – Eur. J. 2024, 30, 202400755.

[72] T. V. Plakhova , A. Y. Romanchuk , A. D. Konyukhova , I. F. Seregina , A. E. Baranchikov , R. D. Svetogorov , M. W. Terban , V. K. Ivanov , S. N. Kalmykov , Environ. Sci. Nano 2024, 11, 3551.

[73] P. Estevenon , L. Amidani , S. Bauters , C. Tamain , M. Bodensteiner , F. Meurer , C. Hennig , G. Vaughan , T. Dumas , K. O. Kvashnina , Chem. Mater. 2023, 35, 1723.

[74] H. J. Sokol , A. M. Ebrahim , S. Caratzoulas , A. I. Frenkel , J. A. Valla , J. Phys. Chem. C 2022, 126, 1496.

[75] S. M. Butorin , K. O. Kvashnina , J. R. Vegelius , D. Meyer , D. K. Shuh , Proc. Natl. Acad. Sci. USA 2016, 113, 8093. 27370799 10.1073/pnas.1601741113 PMC4961124

[76] L. M. Moreau , E. Lapsheva , J. I. Amaro‐Estrada , M. R. Gau , P. J. Carroll , B. C. Manor , Y. Qiao , Q. Yang , W. W. Lukens , D. Sokaras , E. J. Schelter , L. Maron , C. H. Booth , Chem. Sci. 2022, 13, 1759. 35282640 10.1039/d1sc06623d PMC8827158

[77] S. Yang , W. Zhang , G. Pan , J. Chen , J. Deng , K. Chen , X. Xie , D. Han , M. Dai , L. Niu , Angew. Chem., Int. Ed. 2023, 62, 202312076. 10.1002/anie.202312076 37667537

[78] S. Yamazaki , T. Matsui , T. Ohashi , Y. Arita , Solid State Ion 2000, 136, 913.

[79] J. Guo , R. Song , Z. Li , D. Pan , H. Xie , Y. Ba , M. Xie , S. Fan , X. Yang , H. Zhang , H. Yu , S. Zhang , J. Du , L. He , L. Wang , Adv. Energy Sustain. Res. 2022, 3, 2200106.

[80] O. V. Safonova , A. Guda , Y. Rusalev , R. Kopelent , G. Smolentsev , W. Y. Teoh , J. A. van Bokhoven , M. Nachtegaal , ACS Catal. 2020, 10, 4692.

[81] S. Chen , L. Costley‐Wood , I. Lezcano‐Gonzalez , E. Campbell , Z. Weng , M. Asunción Molina , Y. Wu , A. M. Beale , Appl. Catal. B Environ. Energy 2025, 366, 125029.

[82] R. Hissariya , R. Sharma , S. K. Mishra , J. Phys. Chem. Solids 2023, 181, 111549.

[83] W. Li , J. Lv , D. Liu , W. Cai , X. Chen , Q. Huang , L. Wang , B. Wang , Chem. Mater. 2023, 35, 3892.

[84] P. Hongmanorom , J. Ashok , P. Chirawatkul , S. Kawi , Appl. Catal. B Environ. 2021, 297, 120454.

[85] R. Khatun , N. Siddiqui , R. Singh Pal , S. Bhandari , T. Suvra Khan , S. Singh , M. Kumar Poddar , C. Samanta , R. Bal , Catal. Sci. Technol. 2023, 13, 6431.

[86] R. Khatun , R. Singh Pal , K. Bhati , A. Chandra Kothari , S. Singh , N. Siddiqui , S. Rana , R. Bal , RSC Sustain. 2025, 3, 844.

[87] G. I. Siakavelas , N. D. Charisiou , A. AlKhoori , S. AlKhoori , V. Sebastian , S. J. Hinder , M. A. Baker , I. V. Yentekakis , K. Polychronopoulou , M. A. Goula , J. CO2 Util. 2021, 51, 101618.

[88] K. Liu , X. Xu , J. Xu , X. Fang , L. Liu , X. Wang , J. CO2 Util. 2020, 38, 113.

[89] A. Cárdenas‐Arenas , A. Quindimil , A. Davó‐Quiñonero , E. Bailón‐García , D. Lozano‐Castelló , U. De‐La‐Torre , B. Pereda‐Ayo , J. A. González‐Marcos , J. R. González‐Velasco , A. Bueno‐López , Appl. Catal. B Environ. 2020, 265, 118538.

[90] A. Cárdenas‐Arenas , A. Quindimil , A. Davó‐Quiñonero , E. Bailón‐García , D. Lozano‐Castelló , U. De‐La‐Torre , B. Pereda‐Ayo , J. A. González‐Marcos , J. R. González‐Velasco , A. Bueno‐López , Appl. Mater. Today 2020, 19, 100591.

[91] Y. Xu , R. Chen , H. Lin , Q. Lv , B. Liu , L. Wu , L. Tan , Y. Dai , X. Zong , Y. Tang , J. Catal. 2024, 435, 115545.

[92] Y. Sun , T. Wu , Z. Bao , J. Moon , Z. Huang , Z. Chen , H. Chen , M. Li , Z. Yang , M. Chi , T. J. Toops , Z. Wu , D. Jiang , J. Liu , S. Dai , ACS Cent. Sci. 2022, 8, 1081. 36032771 10.1021/acscentsci.2c00340 PMC9413438

[93] L. Wang , Q. Yang , M. Huo , D. Lu , Y. Gao , Y. Chen , H. Xu , Adv. Mater. 2021, 33, 2100150. 10.1002/adma.202100150 34146359

[94] K. Kenyotha , P. Kidkhunthod , Y. Poo‐arporn , K. C. Chanapattharapol , J. Phys. Chem. Solids 2024, 190, 112009.

