# Supplementary material for: The Dual‐Active‐Site Catalysts Containing Atomically Dispersed Pr3+ with Ni/CeO2 for CO2 Hydrogenation to Methane
Source: Small. 2025 Jun 11;21(32):2504707. doi: 10.1002/smll.202504707 (PMC12366274; doi:10.1002/smll.202504707)
Supplement: Supplementary file 1 — Supporting Information [file SMLL-21-2504707-s001.docx]

**Supporting information**

**The Dual-active-site Catalysts containing Atomically Dispersed Pr^3+^ with Ni/CeO_2_ for CO_2_ hydrogenation to Methane**

Neha Choudhary^a^, Navdeep Srivastava^b^, Harshini V. Annadata^c^, Biplab Ghosh^c^, Patrick Da Costa*^a^

^a^Institut Jean Le Rond D’Alembert, Sorbonne Université, CNRS UMR 7190, 2 Place de la Gare de Ceinture, 78210 Saint-Cyr-L’Ecole, France

^b^Department of Chemistry, Indian Institute of Technology-Indore, Simrol, Khandwa Road, 453552, India

^c^Beamline Development and Application Section, Bhabha Atomic Research Center, Mumbai 400085, India

*Email: [patrick.da_costa@upmc.fr](mailto:patrick.da_costa@upmc.fr)

**1. Experimental Section**

*1.1 Materials*

All required metal salts i.e., nickel (II) nitrate hexahydrate (Ni(NO_3_)_2_⋅6H_2_O) ≥99% pure, praseodymium nitrate hexahydrate (Pr(NO_3_)_3_.6H_2_O) ≥99% pure and cerium (III) nitrate hexahydrate (Ce(NO_3_)_3_.6H_2_O) ≥99.9% pure were purchased from Sigma-Aldrich. Additionally, ammonium solution, ethanol and acetone were purchased from Merck all the chemicals were used without further purification.

*1.2 Catalyst synthesis*

*1.2.1 Synthesis of Ni-Pr/CeO_2_ SACs*

For preparing Ni-Pr/CeO_2_ catalyst, in a round bottom flask, praseodymium nitrate hexahydrate and cerium nitrate hexahydrate were first dissolved in 60 mL water in a 1:9 molar ratio and let it stir until a clear solution observed at room temperature. After that, ammonia solution was dropwise added to the above solution to maintain the pH 9 and let it stir at room temperature. After 2 hours, the solution was washed to pH 7 and centrifuged several times. The obtained precipitates were then calcined at 600 ℃ at 5℃/min ramping time for 2 hours and obtained material named as atomically dispersed Pr/CeO_2_ catalyst.

Afterward, in a beaker, 1.34 gm of Ni(NO_3_)_2_.6H_2_O was dissolved in 20 mL ethanol and stirred for 15 minutes. In a clear solution of nickel nitrate, 2.7 gm of Pr/CeO_2_ SAC was dispersed and heated at 60 ℃ temperature in water bath until a thick paste formed. After this, the obtained paste was dried in oven at 100 ℃ and calcined at 600 ℃ at 5℃/min ramping time for 2 hours and named as Ni-Pr/CeO_2_ catalyst.

*1.2.2 Synthesis of Ni/CeO_2_*

Initially, CeO_2_ was prepared using the co-precipitation method where cerium nitrate Ce(NO_3_)_3_.6H_2_O in required amount of water was dissolved and stirred until the solution was clear. After that, ammonia solution was added dropwise to the above aqueous solution with continuous stirring to maintain the pH 9. After maintaining the pH, the solution was stirred for another 2 hours. During this step, the colourless solution turned milky. After 2 hours, the solution was centrifuged and washed with acetone and water till pH 7 and then dried overnight in oven at 100 ℃. Further, obtained precipitates are calcined at 600 ℃ at 5℃/min ramping time for 2 hours and named as CeO_2_ or ceria.

Further, in a round bottom flask, 500 gm of nickel nitrate was dispersed in 10 mL ethanol and added to above synthesized 1 gm of ceria solid powder and heated at 60 ℃ in water bath till the evaporation of the solution. After that, the obtained thick paste was collected and dried in oven at 100 ℃ overnight and further calcined at 600 ℃ at 5℃/min ramping time for 2 hours and named as Ni/CeO_2_ catalyst.

*1.2.3 Synthesis of Ni-Pr supported over CeO_2_ (NiPr/CeO_2_-imp)*

For comparison, NiPr/CeO_2_ impregnated catalyst was also prepared where Ni and Pr were impregnated over CeO_2_ support. For synthesizing this, 0.75 gm of nickel nitrate hexahydrate and 0.5 gm of praseodymium nitrate hexahydrate were dissolved together in an adequate amount of water using a magnetic stirrer at room temperature. This aqueous solution of metal salts was then added to pre-synthesized 1.5 gm of ceria and stirred for 1 hour in a round bottom flask. After 1 hour, the solution was dried under reduced pressure and obtained precipitates were dried overnight in oven at 100 ℃ and then calcined at 600 ℃ for 2 hours. The obtained solid powder was named as NiPr/CeO_2_-impregnated or NiPr/CeO_2_-imp catalyst.

*1.3 Catalytic measurement*

The catalytic test was performed in a fixed bed tubular quartz U-shaped reactor equipped with K-type thermocouple on the outer wall of the reactor in the heating furnace to estimate the temperature during the process. The catalyst was packed with quartz wool from the lower side to prevent the catalyst loss with gas flow. Initially, all the catalysts were reduced at 400 ℃ temperature under the flow of 5% H_2_/Ar gas with 100 mL/min flow rate for 1 hour considering gas hour space velocity (GHSV) 25000 h^-1^. After reduction, the temperature was cooled down to 250 ℃, and then the mixture of Ar/CO_2_/H_2_ = 25:15:60 was purged with a total flow of 100 mL/min with increasing the temperature from 250 ℃ to 450 ℃ with 10 ℃/min ramping temperature. At each temperature step, the sample was kept for at least 30 minutes. The conversion and selectivity of the desired product were estimated using an online gas chromatogram Agilent Varian GC4900 equipped with a thermal conductivity detector (TCD). For equilibrium calculation, the amount of CO_2_:H_2_ was considered in 1:4 ratio with 1 bar pressure from 100 to 450 ℃ temperature range. The conversion (X_CO2_) and selectivity (S_CH4_) were calculated based on the following formulas:

CO_2_ conversion (%) $X$CO_2_ = $\frac{\left[ {CO}_{2} \right]_{\mathrm{in}}-\left[ {CO}_{2} \right]_{\mathrm{out}}}{\left[ {CO}_{2} \right]_{\mathrm{in}}}\times100\%$

CH_4_ selectivity (%) $S$CH_4_ = $\frac{\left[ {CH}_{4} \right]_{\mathrm{out}}}{\left[ {CH}_{4} \right]_{\mathrm{out}}+\left[ \mathrm{CO} \right]_{\mathrm{out}}}\times100\%$

STY_CH4_ = $\frac{\mathrm{SV} X \left[ {CO}_{2} \right]_{in} X x_{CO2} X S_{CH4}}{V_{m}}$ = $\frac{\left[ {CH}_{4} \right]_{\mathrm{out}}}{\left[ {CH}_{4} \right]_{\mathrm{out}}+\left[ \mathrm{CO} \right]_{\mathrm{out}}}$

In the above-mentioned reactions, in and out represent the concentration of gas flow rate in the inlet and outlet. $x$CO_2_ and $x$CH_4_ represents CO_2_ conversion and methane selectivity, respectively. STY represents the space-time yield for methane, SV represents space velocity (h^-1^), V_m_ is the molar volume of specific temperature (mL/mol).

*1.4 Physicochemical characterizations*

Powder x-ray diffraction (PXRD) measurements of the fresh and reduced catalysts were recorded on Bruker D8_Advance diffractometer, equipped with a LynxEye XE-T detector, and Cu anticathode (Cu Kα radiation (0.154 nm)) with a sampler at the UCCS, University of Lille, France. To analyze the data, recorded XRD patterns were compared with the database of the Joint Committee on Powder Diffraction Standards (JCPDS) and the crystallite size was calculated using Debye Scherrer equation. It is a Bruker D8_Advance diffractometer, equipped with a LynxEye XE-T detector, copper anticathode, with a sampler.

N_2_ adsorption-desorption study to analyze the surface area, pore size and pore volume was performed using the Belsorp Mini II from BEL Japan. For this, the material was outgassed for 2 hours at 300 ℃. The analysis was performed at 77 K temperature at 1 bar pressure. The surface area was estimated with the Brunauer–Emmett–Teller (BET) method, and pore size and volume were estimated by the Barrett–Joyner–Halenda (BJH) method using BEL master software. Elemental analysis was done by inductively coupled plasma–optical emission spectrometry (ICP-OES) technique using Agilent 5110 at IC2MP, University of Poitiers, France.

Temperature-programmed reduction (H₂-TPR), CO_2_ temperature-programmed desorption (CO_2_-TPD) and H_2_ temperature-programmed desorption (H_2_-TPD) experiments were conducted using a BELCAT-M instrument (BEL Japan, Japan) equipped with a thermal conductivity detector (TCD). All these tests were performed using quartz tube equipped with a thermocouple in a furnace. For the H_2_-TPR study, 55-60 mg of catalyst was outgassed at 100 °C for 1 hour to remove the moisture under helium flow (50 mL/min) with 10 ℃/min ramping temperature, and then the sample was cooled down to 50 °C, and the reactor was flushed with He for another 20 minutes to remove physically adsorbed hydrogen. Further, catalyst was then heated with 10 °C/min heating rate from 50 to 700 °C under 5% H_2_:Ar (50 mL/min flow). The estimation of consumed H_2_ was done in terms of TCD signal values with increasing temperature. Just after H_2_-TPR, the samples were directly utilized for CO_2_-TPD analysis. Initially, the samples were treated under He for 20 minutes with increasing the temperature from room temperature (RT) to 100 ℃, and then the gas flow was switched to 10% CO_2_/He for 1 hour to adsorb the CO_2_ on the surface of catalysts. After this, the sample was cooled down to 50 ℃ under 10% CO_2_/He flow (50 mL/min) and then switched to He flow and kept for 20 minutes to remove weakly adsorbed CO_2_ from the surface. Afterward, the analysis was carried out under He flow from 50 to 650 ℃ with 10 ℃/min ramping temperature. The basicity calculation was done by considering the area under the curve with comparison of standard calibrated samples area for precise values. In H_2_-TPD, the fresh catalyst was first reduced under 5% H_2_:Ar gas for 1 hour with increasing the temperature from RT to 400 ℃ with 10 ℃/min heating rate and cooled down to 50 ℃. After that, the catalyst was kept at 50 ℃ for another 1 hour under He flow to remove physically adsorbed hydrogen from the surface. After that, the catalysts were heated under He atmosphere from 50 to 600 ℃ with 10 ℃/min ramping temperature.

Dispersion of single-atom over support was analyzed via Transmission electron microscopy (TEM) analysis using JEOL 1011 microscope (operable at 100 kV) by JEOL LTD, Japan. High-resolution transmission electron microscopy (HR-TEM) and STEM (scanning tunneling electron microscopy), and Energy Dispersive X-ray spectroscopy (EDX) were performed using JEM 2100 plus from JEOL, Japan microscope with 200 kV acceleration voltage. For preparing the samples for analysis, the catalyst was dispersed in ethanol and drop-casted over copper grid.

The X-ray Photoelectron Spectroscopy (XPS) analysis of catalysts was recorded using Scient Omicron Multiprobe XPS spectrometer using Auger Electron Spectroscopy (AES) PHI 5000 Versa Prob II, FEI Inc at MNIT Jaipur, India.

X-ray Absorption Spectroscopy (XAS) measurements, including both X-ray Near Edge Structure (XANES) and Extended X-ray Absorption Fine Structure (EXAFS) techniques, were conducted to investigate the impact of Pr doping on the local structure of Ni-Pr/CeO_2_ and Ni/CeO_2_ catalysts. These measurements were performed at the Pr and Ce L₃-edges and the Ni K-edge. The experiments took place at the Energy-Scanning EXAFS beamline (BL-9) of the Indus-2 Synchrotron Source (2.5 GeV, 100 mA) at the Raja Ramanna Centre for Advanced Technology (RRCAT) in Indore, India. This beamline operates within an energy range of 4 keV to 25 keV.

The beamline optics include a Rh/Pt-coated meridional cylindrical mirror for collimation, followed by monochromatization using a Si (111) double crystal monochromator (DCM) with a lattice spacing of 6.2709 Å. The second crystal of the DCM, a sagittal cylinder, enables horizontal focusing, while vertical focusing at the sample position is achieved using a Rh/Pt-coated bendable post mirror. To minimize higher harmonic content in the X-ray beam, the second crystal of the DCM is detuned. For the present study, XAS data were collected in transmission mode at the Ce L_3_-edge and in fluorescence mode at the Pr L_3_-edge and Ni K-edge.

For the transmission measurements, three ionization chambers (each 300 mm in length) were utilized for data acquisition. The first ionization chamber was used to measure the incident flux (I₀), the second to record the transmitted flux (Iₜ), and the third to obtain the XAS spectrum of a reference metal foil for energy calibration. To optimize the signal-to-noise ratio, appropriate gas mixtures and pressures were selected, ensuring 10–20% absorption in the first ionization chamber and 70–90% absorption in the second. The absorption coefficient μ is obtained using the relation:

$I_{T} =I_{0} e^{-\mu x}$ (S1)

where *x* corresponds to the thickness of the absorber. In fluorescence mode, a silicon drift detector (SDD) is employed to measure the fluorescence intensity (I_f_), while an ionization chamber is used to record the incident flux (I_o_). The absorption coefficient is obtained using the relation μ= I_f_/I_0_.

The local structural environment around the absorbing atom is determined through the quantitative analysis of EXAFS spectra. To account for oscillations in the absorption spectra, the data is transformed into an absorption function, which is defined as follows:

$\chi(E)=\frac{\mu(E)-\mu_{0}(E)}{\begin{matrix} \Delta\mu_{0}(E_{0}) \\ \end{matrix}}$ (S2)

where *E_0_* is absorption edge energy, $\mu_{0}(E_{0})$ is the bare atom background and $\Delta\mu_{0}(E_{0})$is the step in $\mu(E)$value at the absorption edge. The energy-dependent absorption coefficient $\chi(E)$ has been converted to the wave number-dependent absorption coefficient $\chi(k)$using the relation,

(S3)

Here, m represents the electron mass. The function $\chi(k)$) is weighted by *k^2^* to enhance oscillations at higher k values, and the *χ(k)k^2^* functions are subjected to Fourier transformation to generate$\chi(R)$ versus R plots, representing the real distances from the absorbing atom's center.

EXAFS data analysis was conducted using the suite of programs available in the Demeter software package. This process involved background subtraction and Fourier transformation of the absorption spectra to obtain χ(R) versus R plots using ATHENA. Theoretical EXAFS spectra were then generated based on an assumed crystallographic structure, followed by fitting the experimental data with the theoretical model using ARTEMIS. For background removal and fitting, different modules of the Demeter with Strawberry Perl (version 0.9.25) software were used.^[1]^ Morlet wavelet transform as employed in the EvAX 5.1 code^[2]^  was used in the *k*-space range of 3-10 Å^−1^, and *R*-space range of 0-6 Å.

**Table S1. Elemental analysis of all the catalysts from ICP-OES analysis**

| Catalysts | Element (Mass %) | | |
| --- | --- | --- | --- |
|  | **Ni** | **Pr** | **Ce** |
| Pr/CeO_2_ | - | 7.7 | 71.8 |
| Ni/CeO_2_ | 7.9 | - | 71.0 |
| Ni-Pr/CeO_2_ | 8.6 | 6.7 | 63.3 |
| NiPr/CeO_2_-imp | 7.5 | 8 | 64.3 |

**Table S2.** Crystallite size of fresh and reduced catalysts

| Catalyst | Fresh (nm) | | |  | Reduced (nm) | |
| --- | --- | --- | --- | --- | --- | --- |
|  | d_(200)_ NiO^a^ | d_(111)_ NiO^b^ | d_(111)_ CeO_2_ |  | d_(111)_ Ni(0) | d_(111)_ CeO_2_ |
| Pr/CeO_2_ | - | - | 15.75 |  | - | 16.60 |
| Ni-Pr/CeO_2_ | 27.06 | 20.85 | 15.57 |  | 26.38 | 15.75 |
| Ni/CeO_2_ | 22.33 | 22.46 | 16.07 |  | 29.22 | 16.96 |

^a^Calculation of crystallite size for the most intense peak at (200) plane at 2θ = 43.6º.

^b^Calculation of (111) plane for comparison of the fresh catalyst with the reduced catalyst at 2θ = 37.36º and 44.4º, respectively.


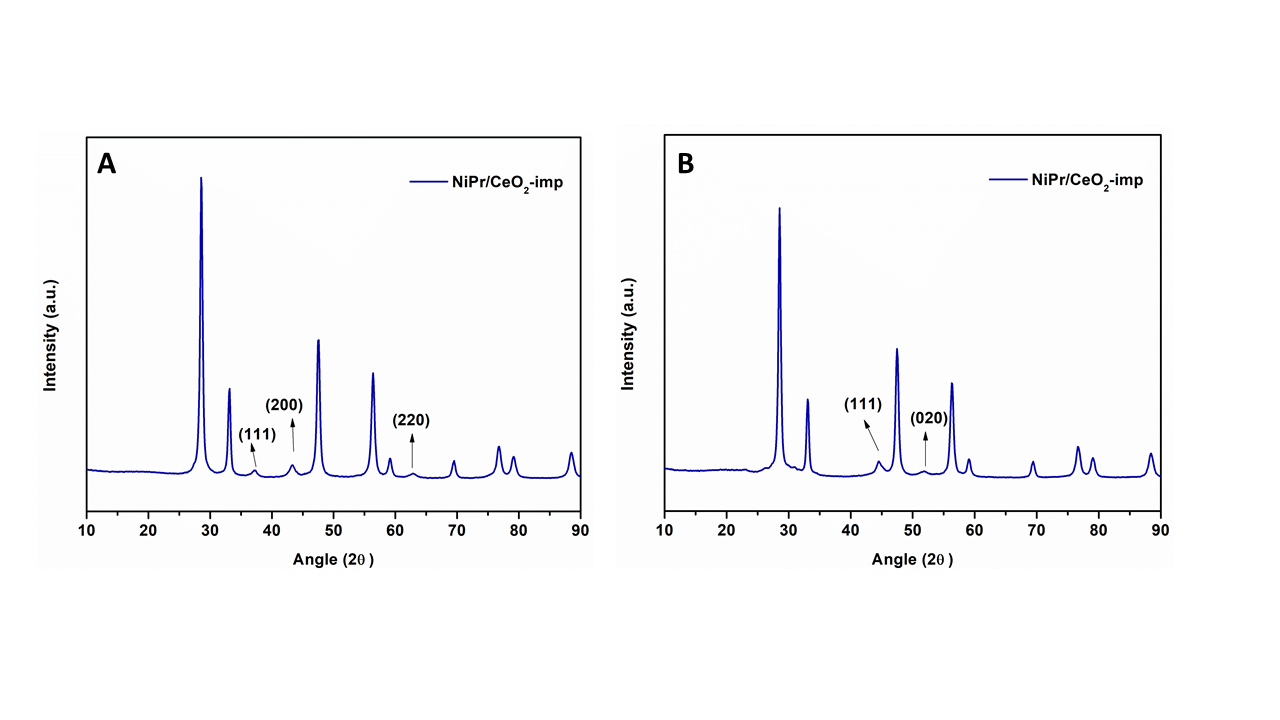


**Fig. S1.** PXRD spectra of NiPr/CeO_2_-imp catalyst **(A)** fresh and **(B)** reduced.


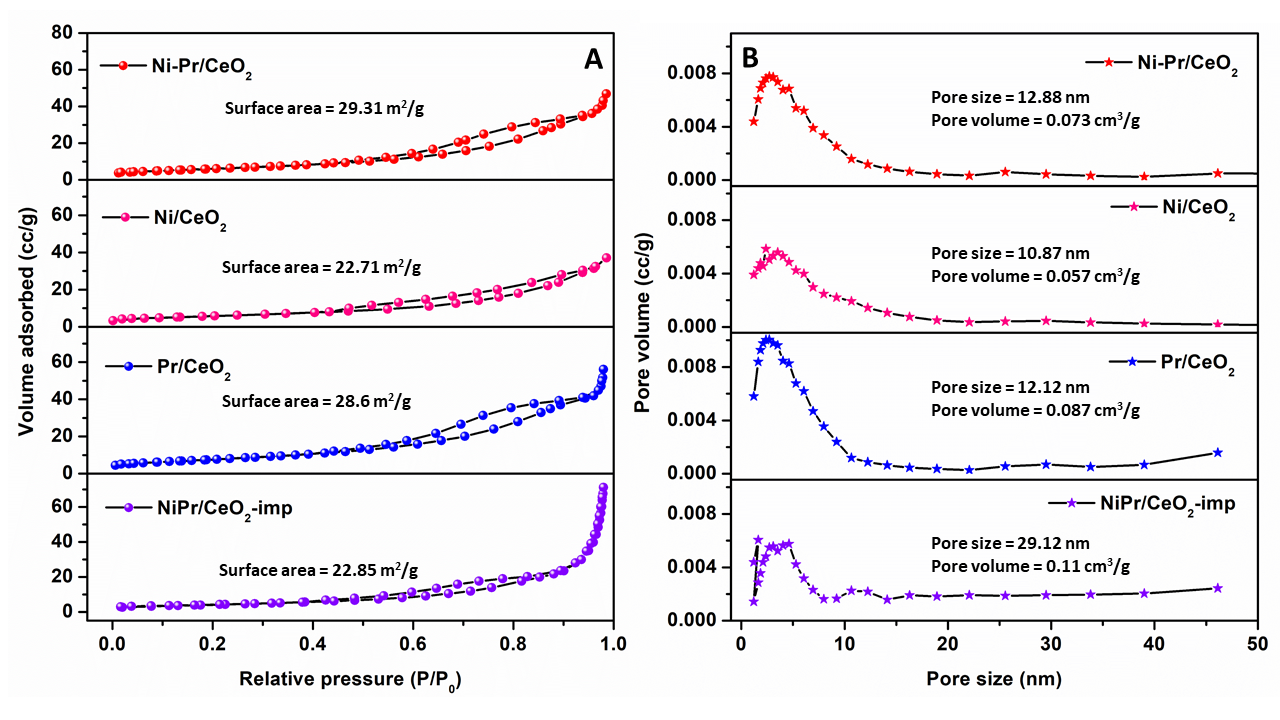


**Fig. S2.** N_2_ adsorption-desorption studies for (**A**) surface area and (**B**) pore size distribution of all catalysts.


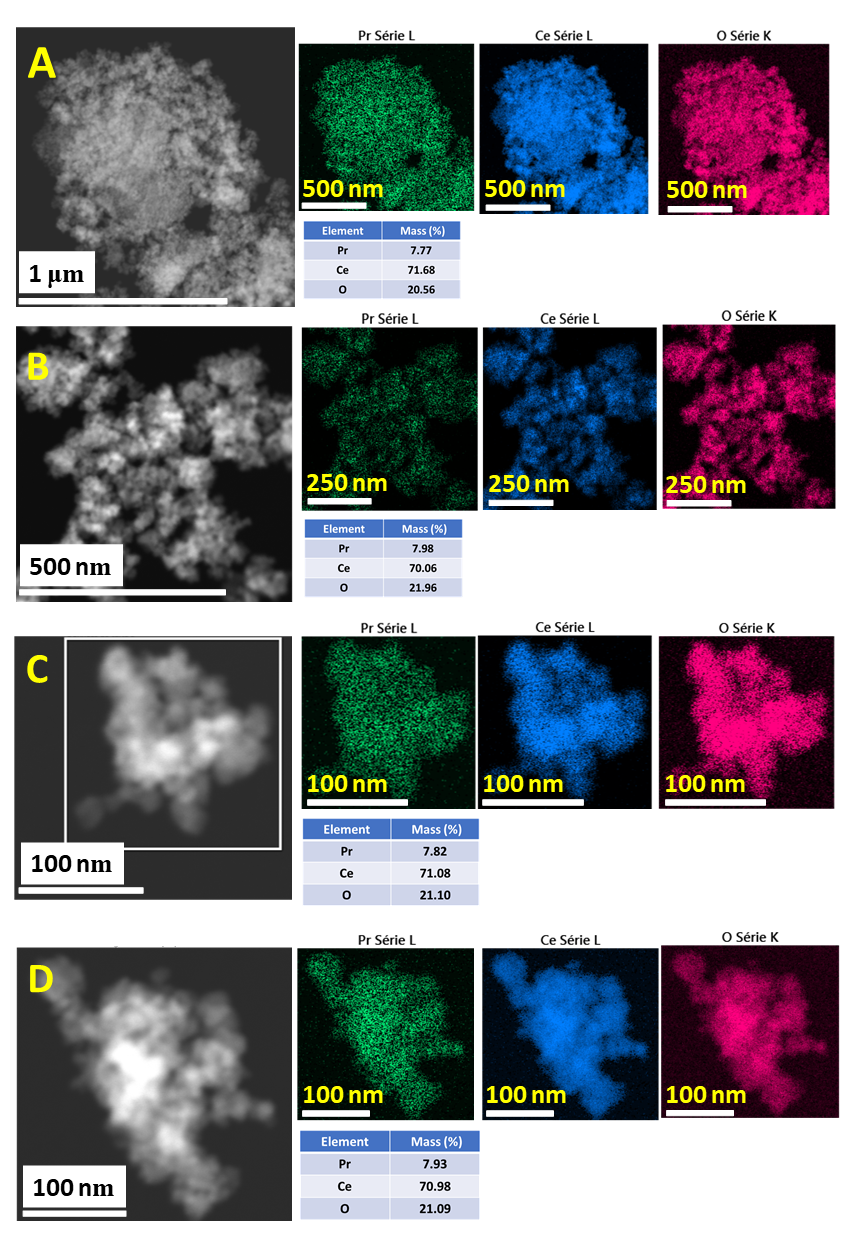


**Fig. S3.** HAADF imaging and STEM mapping of Praseodymium, Cerium, and Oxygen elements of Pr/CeO_2_ catalyst at different regions at higher magnifications with scale bars of (**A**) 500 nm, (**B**) 250 nm, (**C**) 100 nm, and (**D**) 100 nm.

**
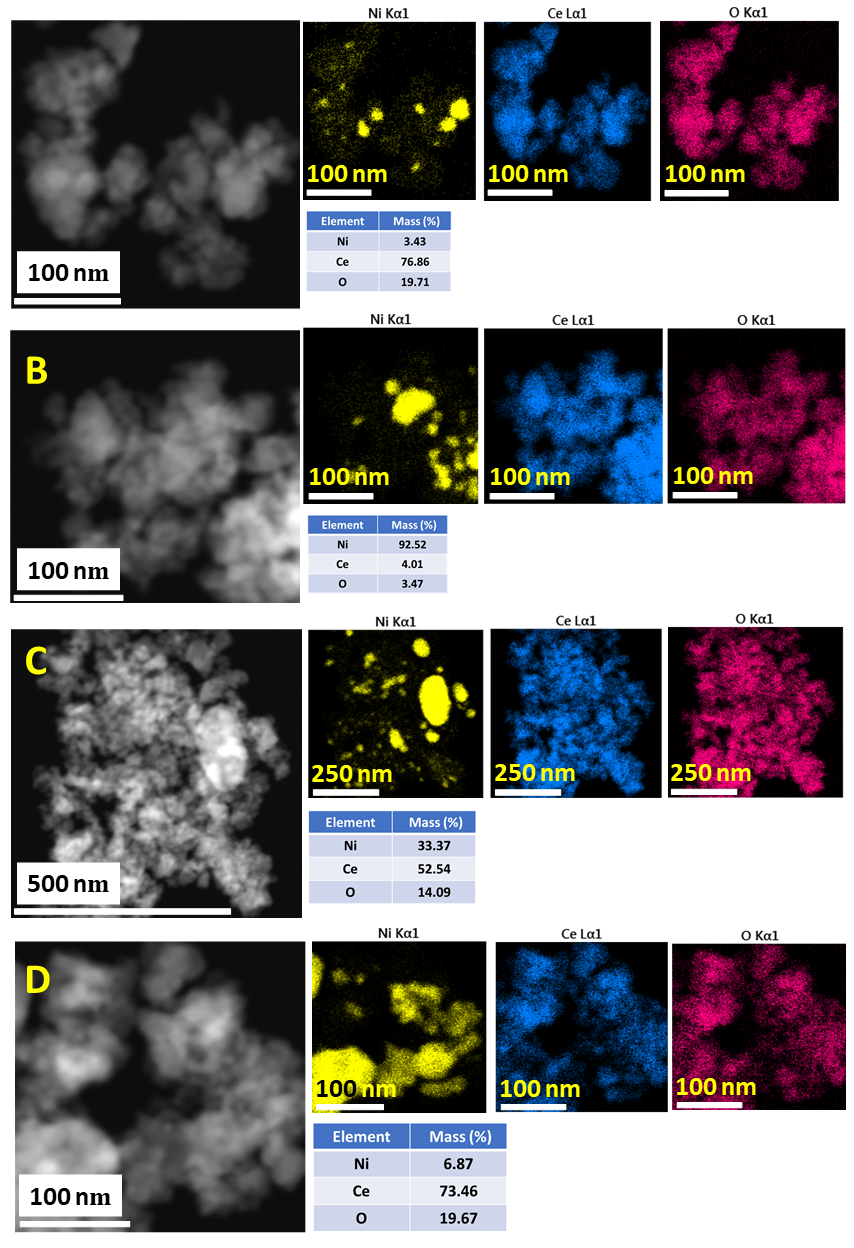
**

**Fig. S4.** HAADF imaging and STEM mapping of Nickel, Cerium, and Oxygen elements of Ni/CeO_2_ catalyst at different regions at higher magnifications with scale bars of (**A**) 100 nm, (**B**) 100 nm, (**C**) 250 nm, and (**D**) 100 nm.

**
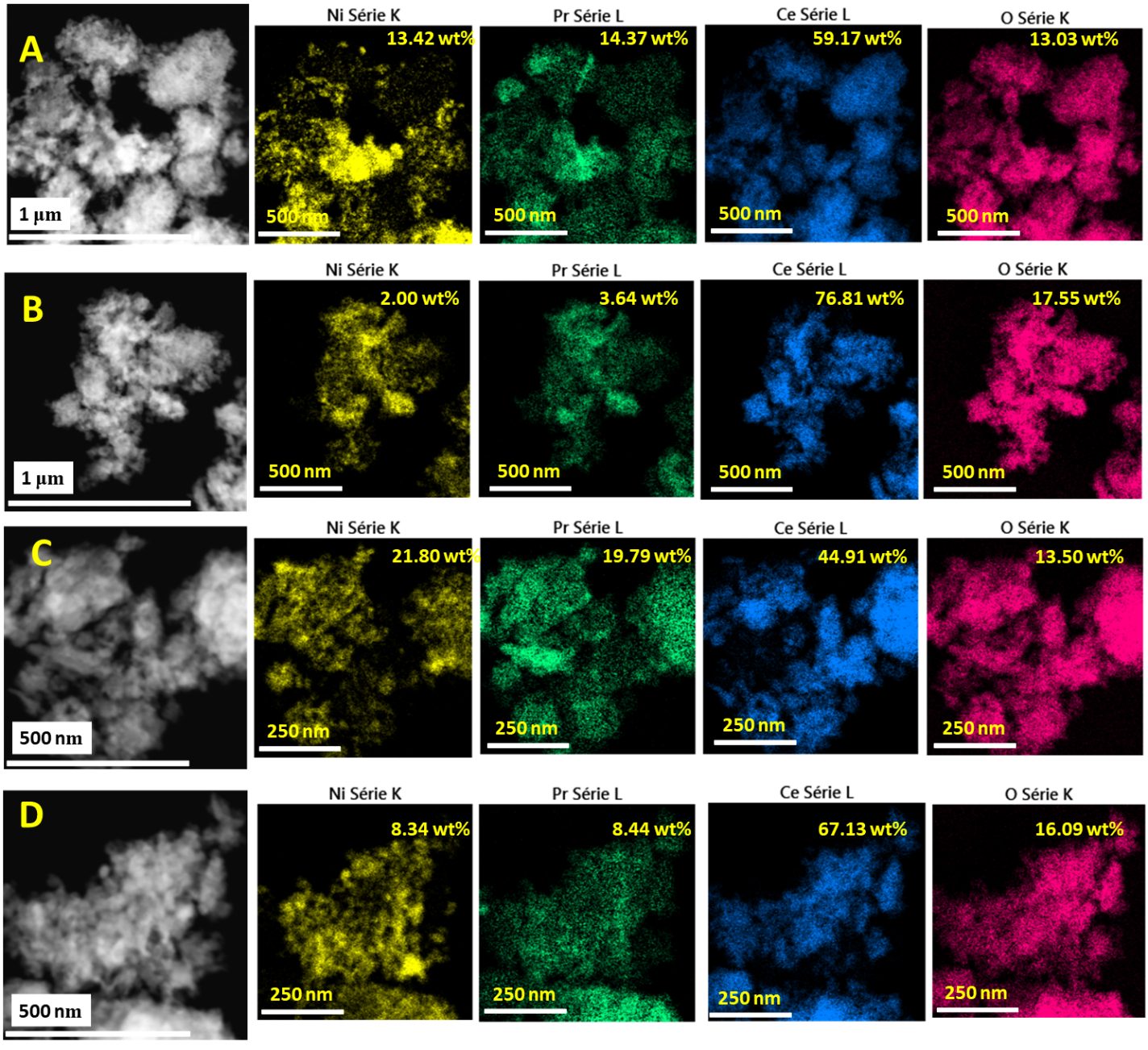
**

**Fig. S5.** HAADF imaging and STEM mapping of Nickel, Praseodymium, Cerium and Oxygen elements of NiPr/CeO_2_-imp catalyst at different regions at higher magnifications with scale bars of (**A**) 500 nm, (**B**) 500 nm, (**C**) 250 nm, and (**D**) 250 nm.

**
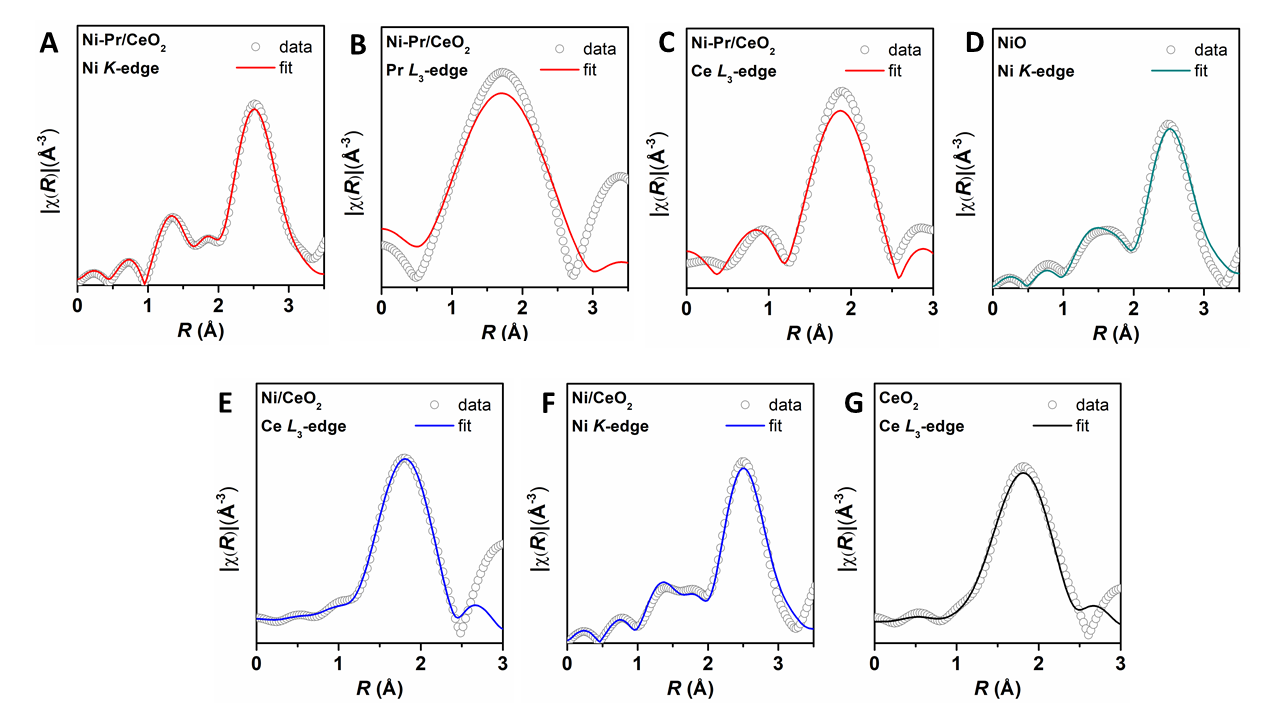
**

**Fig. S6.** First shell fits of Ni-Pr/CeO_2_ for Ni K-edge, Ce L_3_-edge and Pr L_3_-edge (**A-C**) and NiO reference for Ni K-edge (**D**) and Ni/CeO_2_ for Ni K-edge, Ce L_3_-edge (**E-F**) and CeO_2_ reference for Ce L_3_-edge (**G**).

**Table S3**. Comparative study of previously reported catalyst for methanation at 300 ℃ temperature

| S. No. | Catalyst | Synthetic method | Conv. (%) | Sel. (%) | WHSV (ml/g/h) | GHSV (h^-1^) | Ref. |
| --- | --- | --- | --- | --- | --- | --- | --- |
| 1 | Ni/CSG (Ce_0.9_Pr_0.1_O_2-δ_) | Citrate Sol-gel | 46 | 98 | 25000 | - | [3] |
| 2 | Ni/PC (Ce_0.9_Pr_0.1_O_2-δ_) | Pechini synthesis | 33 | 97 | 25000 | - | [3] |
| 3 | Ni/MPC (Ce_0.9_Pr_0.1_O_2-δ_) | Modified Pechini synthesis | 71 | 99 | 25000 | - | [3] |
| 4 | Ni/Pr5Ce | Citrate sol-gel method | 45 | 98 | 25000 | - | [4] |
| 5 | Ni/Pr10Ce | Citrate sol-gel method | 46 | 98 | 25000 | - | [4] |
| 6 | Ni/Pr20Ce | Citrate sol-gel method | 40 | 97 | 25000 | - | [4] |
| 7 | Ni/Pr50Ce | Citrate sol-gel method | 21 | 95 | 25000 | - | [4] |
| 8 | Ni/Pr-Ce | MW with sol-gel | 24 | 100 | 25000 | - | [5] |
| 9 | Ni/La-Pr-CeO_2_ | MW with sol-gel | 21 | 100 | 25000 | - | [6] |
| 10 | Ru/Ce3PrO_x_ | Mechano-chemical + Incipient wetness | ~81 | - | - | 9000 | [7] |
| 11 | **Ni-Pr/CeO_2_** | **Co-precipitation + wetness** | **87** | **100** | **-** | **25000** | **This work** |

**Reference:**

[1] B. Ravel, M. Newville, *J. Synchrotron Radiat.* **2005**, *12*, 537–541.

[2] J. Timoshenko, A. Kuzmin, J. Purans, *J. Phys. Condens. Matter* **2014**, *26*, 055401.

[3] A. I. Tsiotsias, N. D. Charisiou, E. Harkou, S. Hafeez, G. Manos, A. Constantinou, A. G. S. Hussien, A. A. Dabbawala, V. Sebastian, S. J. Hinder, M. A. Baker, K. Polychronopoulou, M. A. Goula, *Appl. Catal. B Environ.* **2022**, *318*, 121836.

[4] A. I. Tsiotsias, N. D. Charisiou, A. AlKhoori, S. Gaber, V. Stolojan, V. Sebastian, B. van der Linden, A. Bansode, S. J. Hinder, M. A. Baker, K. Polychronopoulou, M. A. Goula, *J. Energy Chem.* **2022**, *71*, 547–561.

[5] G. I. Siakavelas, N. D. Charisiou, S. AlKhoori, A. A. AlKhoori, V. Sebastian, S. J. Hinder, M. A. Baker, I. V. Yentekakis, K. Polychronopoulou, M. A. Goula, *Appl. Catal. B Environ.* **2021**, *282*, 119562.

[6] G. I. Siakavelas, N. D. Charisiou, A. AlKhoori, S. AlKhoori, V. Sebastian, S. J. Hinder, M. A. Baker, I. V. Yentekakis, K. Polychronopoulou, M. A. Goula, *J. CO2 Util.* **2021**, *51*, 101618.

[7] S. L. Rodríguez, A. Davó-Quiñonero, J. Juan-Juan, E. Bailón-García, D. Lozano-Castelló, A. Bueno-López, *J. Phys. Chem. C* **2021**, *125*, 12038–12049.
